# Food-Grade By-Products as Sustainable Sources of Phytochemicals: Green Extraction Strategies, Encapsulation Technologies, and Applications in Functional Food Development

**DOI:** 10.3390/ijms27198862

**Published:** 2026-10-05

**Authors:** Marek Kruczek, Patryk Pokorski, Barbara Drygaś, Dorota Gumul

**Affiliations:** 1 Department of Carbohydrate Technology and Cereal Processing, Faculty of Food Technology, University of Agriculture in Krakow, 122 Balicka Street, 30-149 Krakow, Poland; dorota.gumul@urk.edu.pl; 2 Faculty of Health Sciences, Warsaw University of Life Sciences, 02-787 Warsaw, Poland; patryk_pokorski@sggw.edu.pl; 3 Department of Bioenergetics, Food Analysis and Microbiology, University of Rzeszow, 35-601 Rzeszów, Poland; badrygas@ur.edu.pl

**Keywords:** encapsulation, food by-products, green extraction, phytochemicals

## Abstract

Food-industry by-products, including fruit and vegetable pomace, peels, and seeds, are increasingly recognized as sustainable sources of bioactive compounds, particularly phenolic compounds and carotenoids, including anthocyanins and lycopene. These phytochemicals exhibit a range of biological activities; however, their application in food systems is often limited by poor stability, low bioavailability, and susceptibility to environmental degradation. This review summarizes recent advances in the recovery and stabilization of bioactive compounds from food-industry by-products. Particular attention is given to green extraction technologies, including ultrasound-assisted, microwave-assisted, pressurized liquid, and supercritical fluid extraction, which can improve extraction efficiency while reducing solvent consumption and environmental impact. Furthermore, conventional and emerging encapsulation approaches, such as spray drying, ionic gelation, nanoemulsions, Pickering emulsions, liposomes, and lipid nanoparticles, are discussed as strategies to improve the stability, bioaccessibility, and delivery of recovered phytochemicals. The integration of green extraction and encapsulation technologies offers a promising strategy for the valorization of food processing residues and the development of innovative functional food ingredients. Such integrated approaches may contribute to waste reduction, improved resource efficiency, and the implementation of circular economy principles in food production.

## 1. Introduction

The role of diet in the prevention and management of chronic diseases is well established. Adherence to healthy dietary patterns can significantly reduce the risk of developing noncommunicable diseases (NCDs), including obesity, type 2 diabetes, cardiovascular diseases, neurodegenerative disorders, and certain types of cancer [1,2]. Consequently, increasing attention has been directed toward functional foods that provide health benefits beyond basic nutrition. According to the Hippocratic concept, food can play an essential role in maintaining health and preventing disease [3]. Growing consumer awareness and rising global demand for sustainable, health-promoting products have stimulated extensive research into functional foods and their diverse bioactive constituents.

Bioactive compounds comprise a diverse group of naturally occurring substances that exert beneficial physiological effects in humans by modulating key molecular pathways [4,5]. These compounds include, among others, phenolic compounds, carotenoids, alkaloids, bioactive peptides, polysaccharides, omega-3 fatty acids, vitamins, and enzymes. Numerous studies have demonstrated their multifunctional properties, including antioxidant, anti-inflammatory, antimicrobial, immunomodulatory, cardioprotective, neuroprotective, and anticancer activities [6,7,8]. Traditionally, plant materials have been considered the primary source of bioactive compounds; however, industrial side streams, such as food processing by-products (e.g., peels, seeds, and pomace), fungi, microorganisms, microalgae, and animal-derived materials are increasingly recognized as sustainable and high-value alternative sources within the framework of a circular bioeconomy [9,10,11,12,13,14,15,16,17].

In recent years, particular attention has been paid to food-industry by-products as sustainable and economically attractive sources of bioactive compounds. Large quantities of by-products, including fruit and vegetable pomace, peels, seeds, bran, and processing residues, are generated annually by the food industry and often remain underutilized despite their high content of valuable phytochemicals and other bioactive constituents [18,19,20]. The recovery and valorization of these materials are consistent with the principles of the circular economy and contribute to waste reduction, resource efficiency, and the development of sustainable food systems [18,20].

Among the bioactive compounds recovered from food-industry by-products, phenolic compounds, including anthocyanins, and carotenoids, including lycopene, have attracted particular scientific interest because of their potent antioxidant and anti-inflammatory properties and their potential role in the prevention of chronic diseases [21,22,23,24]. Nevertheless, despite their considerable biological potential, the application of bioactive compounds in food systems remains challenging. Many of these compounds exhibit low water solubility, poor chemical stability, and high susceptibility to environmental factors such as light, oxygen, temperature, metal ions, and pH changes. Specifically, factors such as all-E/Z isomerization (characteristic of carotenoids, among other things) and matrix-dependent bioaccessibility can significantly limit the amount of bioactive compounds reaching target tissues [25,26]. Bioaccessibility refers to the fraction of a compound released from the food matrix during gastrointestinal digestion and made available for potential absorption. It should be distinguished from bioavailability, which concerns the fraction that reaches the systemic circulation. Consequently, simulated gastrointestinal digestion can provide evidence of release and bioaccessibility, but does not by itself demonstrate systemic bioavailability. Furthermore, they may undergo degradation during food processing, storage, and gastrointestinal digestion, resulting in reduced bioavailability and diminished biological activity [9,27,28,29]. In addition, the incorporation of bioactive compounds into food products may adversely affect sensory attributes, including increased bitterness and astringency, thereby limiting consumer acceptance [30,31,32].

To overcome these limitations, considerable research efforts have focused on the development of effective stabilization strategies. Among these, encapsulation technologies have emerged as one of the most promising approaches for protecting bioactive compounds against environmental degradation, improving their physicochemical stability and bioavailability, masking undesirable sensory characteristics, and enabling controlled release during gastrointestinal digestion [28,33,34]. However, improved encapsulation efficiency, stability, or gastrointestinal release should not be interpreted as evidence of enhanced systemic bioavailability or biological efficacy unless these outcomes are demonstrated using appropriate absorption, pharmacokinetic, in vivo, or clinical approaches. Advanced delivery systems, including Pickering emulsions, liposomes, and solid lipid nanoparticles, offer superior protection and can be tailored for specific food-grade applications. Moreover, the use of appropriate encapsulating materials can significantly enhance the shelf life and functionality of encapsulated compounds [35,36].

In parallel with advances in encapsulation technologies, increasing attention has been devoted to environmentally friendly extraction methods that enable the efficient recovery of bioactive compounds from food-industry by-products while minimizing the use of hazardous solvents and reducing environmental impact. Technologies such as ultrasound-assisted (UAE), microwave-assisted (MAE), and supercritical fluid extraction (SFE) have demonstrated high efficiency in recovering heat-sensitive and non-polar bioactives using food-grade solvents [37,38,39,40]. Importantly, extraction yield or chemical composition alone does not establish bioaccessibility, bioavailability, or physiological efficacy; these outcomes require progressively higher levels of experimental validation. The integration of green extraction technologies with nano- and microencapsulation approaches within a cascade biorefinery framework offers a promising strategy for the sustainable valorization of food processing residues and the development of innovative functional food ingredients [41,42,43].

Consequently, this review provides a structured narrative synthesis of the current literature on food-industry by-products as sustainable reservoirs of bioactive phytochemicals. Particular emphasis is placed on green extraction strategies and advanced nano- and microencapsulation technologies designed to enhance the stability and gastrointestinal bioaccessibility of recovered compounds, alongside their strategic integration into functional food systems.

## 2. Encapsulation Technologies for the Stabilization of Bioactive Compounds

Encapsulation has emerged as a cornerstone strategy for safeguarding the molecular integrity and chemical stability of labile bioactive compounds and enabling their controlled or modulated delivery within complex food matrices. This section systematically delineates the fundamental physicochemical principles underlying core–shell and matrix systems, highlighting their critical role in protecting by-product-derived phytochemicals from environmental stressors and optimizing their functional performance in food-grade applications.

### 2.1. Definition and Mechanism of Action

Encapsulation is a process in which an active substance in a solid, liquid, or gaseous state is entrapped within a food-grade carrier material that forms a protective barrier between the target compound and the surrounding environment. Depending on the particle size, encapsulation systems are generally classified as microencapsulation or nanoencapsulation [44]. The entrapped bioactive substance is commonly referred to as the core, active ingredient, or internal phase, whereas the protective surrounding material is designated as the shell, wall, coating, or matrix, defining whether the delivery platform behaves as a reservoir (core-shell) or a monolithic matrix system [45,46].

The encapsulation process leads to the formation of structures ranging in size from a few nanometers to several hundred micrometers, depending on the encapsulation technique applied and the intended application [47,48]. These systems are designed, among other purposes, to retain and protect bioactive compounds and to enable their release under specific conditions, such as changes in pH, temperature, ionic strength, or enzymatic activity [47]. 

One of the key protective mechanisms of encapsulation is the physical separation of sensitive or labile bioactive compounds from detrimental environmental factors, including oxygen, light, moisture, and elevated temperatures [49,50]. Consequently, the encapsulating barrier can improve the physicochemical stability of the core material, reduce its degradation rate during processing and storage, and enhance bioaccessibility, i.e., the availability of the compound for absorption in the gastrointestinal tract, and, in some cases, also its bioavailability, i.e., the extent of actual absorption and systemic availability [46,51]. Furthermore, encapsulation can effectively mask undesirable sensory attributes, such as the bitterness and astringency of recovered phytochemicals [46,52,53], while enabling controlled or stimuli-responsive release in the gastrointestinal tract in response to physiological factors such as pH gradients, enzymatic activity, or microbial degradation [27,28,53,54,55].

Depending on the selected encapsulation technique and the physicochemical properties of both the core and wall materials, the resulting delivery platforms can be designed in various structural architectures. These include reservoir-type microcapsules, monolithic microspheres, nanoparticles, nanoemulsions, liposomes, and molecular inclusion complexes [46,47,53,56]. Consequently, tailoring these micro- and nano-carriers represents a pivotal strategy for enhancing the thermodynamic stability, preserving the biological functionality, and modulating the release kinetics of sensitive bioactive compounds—such as by-product-derived polyphenols or probiotics—intended for high-value applications in the food, pharmaceutical, cosmetic, and nutraceutical industries [46,51,53].

### 2.2. Application of Encapsulation in the Food Industry

In recent years, encapsulation technologies have attracted considerable attention in the food sector, closely reflecting the growing demand for functional foods enriched with bioactive ingredients [2]. These technologies are particularly useful for compounds characterized by limited physicochemical stability, low aqueous solubility, high susceptibility to thermal, oxidative, or light-induced degradation, as well as limited bioavailability. This group includes, among others, polyphenols, carotenoids, essential oils, polyunsaturated fatty acids, vitamins, enzymes, and probiotic microorganisms [48,57,58].

A particularly important area of application for encapsulation is the valorization of by-products from fruit and vegetable processing, especially pomace, which can represent a concentrated source of polyphenols, anthocyanins, carotenoids, and other bioactive compounds. One example is grape pomace, from which recovered phenolic compounds can subsequently be stabilized through encapsulation using biopolymeric carrier materials such as maltodextrin or pectin [59]. A similar approach has been applied to black carrot pomace, which is rich in anthocyanins and other phenolic compounds, where microencapsulation enables the production of more stable powdered forms with potential application as functional food ingredients [60]. Encapsulation can also be used to protect bioactive compounds recovered from apple pomace, improving their stability during storage and potentially reducing their degradation under gastrointestinal conditions [61].

Several encapsulation techniques, including spray drying, coacervation, extrusion, liposome formation, and nanoemulsification, have been successfully applied in food systems [47]. However, unlike pharmaceutical or veterinary applications, where synthetic carrier systems such as poly(lactic-co-glycolic acid) (PLGA) are widely utilized for targeted delivery [53,62], the selection of encapsulating materials for food products is considerably more restricted due to stringent safety and regulatory requirements [47]. In the European Union, the use of novel or waste-derived encapsulation ingredients is strictly governed by Regulation (EU) 2015/2283 on novel foods, which mandates that any substance not consumed to a significant degree within the Union prior to May 1997 must undergo rigorous safety and toxicological evaluations [51]. In the United States, such ingredients intended for food applications must obtain Generally Recognized As Safe (GRAS) status or comply with formal food additive approval procedures [51]. Consequently, many synthetic polymers and hazardous chemical cross-linking agents, such as glutaraldehyde, commonly employed in pharmaceutical formulations are prohibited in food products due to documented cytotoxicity and lack of biocompatibility [46]. This regulatory bottleneck has shifted the current research paradigm toward food-grade natural polysaccharides, such as alginate, pectin, and starch, which are compatible with clean-label requirements and can safely protect labile core materials [46].

The growing industrial demand for encapsulation technologies closely aligns with current global market trends, highlighting their key role in the transition toward sustainable and circular food systems. While the global microencapsulation market is projected to exceed USD 25 billion by 2030, the upcycled food market—which relies heavily on such stabilization platforms for the valorization of agri-food industry by-products—is estimated to reach USD 79.51 billion by 2034, with a compound annual growth rate (CAGR) of 7.47% [51]. This dynamic commercial expansion underscores the importance of encapsulation as a key platform technology for protecting molecular integrity, reducing degradation rates, and modulating the targeted release of labile bioactive compounds in next-generation functional foods [48,53].

## 3. Food-Processing By-Products: Sources and Phytochemical Composition

The European Union is one of the world’s leading producers of fruits and vegetables. In 2022, vegetable production reached approximately 60 million tonnes, while fruit production exceeded 41 million tonnes, with tomatoes, onions, and carrots ranking among the most important vegetables in terms of production volume, and apples being the dominant pome fruit [63]. Poland plays an important role in the European horticultural sector as one of the major producers of apples and carrots. Fruits and vegetables are rich sources of bioactive phytochemicals, particularly phenolic compounds, which can be used as functional food ingredients [61]. Nevertheless, substantial amounts of food-industry by-products, such as pomace, peels, and seeds generated during the production of juices, beverages, and fermented products, remain underutilized or are treated as waste [51]. For example, tomato processing generates between 4.3 and 10.2 million tonnes of wet pomace worldwide each year [50], while apple pomace represents a substantial by-product biomass composed mainly of peels, pulp, and residual fruit tissues [64]. Similarly, the wine industry generates considerable quantities of grape pomace, accounting for approximately 30% of the mass of processed fruit [59]. At present, a significant proportion of these secondary raw materials is used mainly for energy or animal-feed purposes or remains underutilized [65,66,67]. As a result, their potential as sources of bioactive compounds is not yet fully exploited within the framework of a circular economy [51].

Due to their high content of antioxidant compounds and broad application potential, fruit and vegetable processing by-products, such as pomace, represent promising raw materials for the developing bioeconomy and are consistent with the principles of a circular economy [51,68]. However, their effective valorization requires appropriate quality standards and processing strategies because of the inherent variability in their composition, heterogeneity of the raw material, and high susceptibility to rapid biodegradation [51,69]. Bioactive-rich fractions obtained from these materials, including phenolic-rich extracts and dietary fiber preparations, have considerable potential for use as high-value functional food ingredients [59,70].

Global apple production exceeds 70 million tons annually, generating substantial amounts of processing by-products. The predominant by-product is apple pomace, which consists mainly of peel and pulp (approximately 95%), together with smaller proportions of seeds and stems [64]. Apple pomace is a valuable source of bioactive compounds, including phenolic compounds, dietary fiber, and minerals, which supports its potential use in food, pharmaceutical, and bioenergy applications [71]. Its incorporation into food products, including bakery and confectionery products, dairy products, and meat formulations, may improve their nutritional composition and functional properties [64]. Apple pomace is characterized by a diverse polyphenolic profile comprising several chemical classes, including phenolic acids, flavonols, anthocyanins, dihydrochalcones, and flavan-3-ols. Phloridzin, chlorogenic acid, and quercetin glycosides are among the major phenolic compounds reported in apple tissues and pomace [72]. Because conventional juice processing does not completely extract these compounds, a considerable proportion of phenolic constituents remains in the pomace after pressing, particularly in the peel fraction [73].

Phenolic compounds recovered from apple pomace have attracted considerable interest because of their potential biological activities. Apple-derived phenolic extracts have shown DNA-protective effects and modulation of intestinal barrier function in cell-based models, supporting further investigation of this by-product as a source of functional ingredients [74]. These in vitro findings do not, however, establish physiological benefits following dietary consumption. The extraction strategies used to recover phytochemicals from these materials are discussed in Section 5, while their stabilization and delivery through encapsulation are evaluated in Section 6.

## 4. Types of Encapsulation Techniques

Bioactive compounds recovered from food-industry by-products, including phenolic compounds, carotenoids, and essential oils, often exhibit limited stability during processing and storage, as well as low bioaccessibility and poor compatibility with food matrices [49]. To bypass these technological bottlenecks, encapsulation technologies have emerged as indispensable tools for protecting these labile compounds against environmental degradation, enhancing their biological and technological functionality, and facilitating their seamless incorporation into functional food systems [47,51,54].

Since no single encapsulation technique is universally suitable for all bioactive compounds and functional food systems, a wide range of delivery platforms has been developed. These include conventional approaches such as ionic gelation, extrusion, spray drying, and freeze drying, as well as more advanced lipid-based, molecular, and multilayer systems. The selection of an appropriate encapsulation strategy depends on several factors, including the physicochemical properties of the bioactive core, the desired release profile, such as controlled or stimuli-responsive release, the processing conditions, and the characteristics of the target food matrix. Accordingly, tailored micro- and nanoscale delivery systems play an important role in improving the stability and delivery of sensitive phytochemicals recovered from food-processing by-products. Table 1 summarizes the main principles, advantages, limitations, and typical applications of the key encapsulation techniques used in the development of functional foods.

### 4.1. Gelation- and Extrusion-Based Techniques

Gelation- and extrusion-based techniques are among the most widely applied encapsulation approaches for food bioactive compounds because they operate under mild conditions and are particularly suitable for thermolabile phytochemicals. These methods are commonly used for the encapsulation of phenolic compounds, anthocyanins, carotenoids, probiotics, and essential oils, as they enable the production of biocompatible and biodegradable carriers with controlled release properties [47,75].

Ionic gelation is one of the most extensively studied techniques and is primarily based on the cross-linking of anionic polysaccharides, particularly alginate, in the presence of multivalent cations such as Ca^2+^. Owing to its simplicity and the use of food-grade materials, this technique has been widely employed for the stabilization of phytochemicals recovered from agro-industrial by-products [76].

Several studies have demonstrated the potential of ionic gelation for improving the retention and stability of phenolic compound-rich extracts. For example, alginate–pectin microcapsules containing anthocyanins exhibited improved encapsulation efficiency and enhanced protection under simulated gastrointestinal conditions, enabling controlled release in the intestinal environment [76,77]. Similarly, the incorporation of pullulan into alginate matrices improved the retention of selected phenolic compounds through interactions between the encapsulated compounds and the polymer network [61]. Ionic gelation-based systems have also been successfully applied to probiotic encapsulation. Rao et al. [78] developed calcium pectin-, chitosan–calcium pectin-, and sodium alginate–pectin–whey-based microcapsules for probiotic delivery, demonstrating improved resistance to gastrointestinal stresses and controlled intestinal release. Similarly, Najafpour et al. [79] developed alginate beads incorporating gellan fluid gel for the encapsulation of *Limosilactobacillus reuteri*. These findings further illustrate the versatility of ionically cross-linked polysaccharide matrices for protecting both bioactive compounds and probiotic microorganisms.

Extrusion techniques have also been successfully applied for the encapsulation of bioactive compounds derived from food processing residues. In the microencapsulation of grape pomace extracts, electrostatic extrusion enabled the production of uniform particles with encapsulation efficiencies of approximately 20–30%, depending on capsule size and processing conditions [67]. 

To improve storage stability and facilitate incorporation into food products, gelled particles are frequently subjected to subsequent drying processes. Among these, freeze-drying has been shown to effectively preserve the antioxidant properties and phenolic compound content of encapsulated extracts, particularly in larger capsules containing grape pomace bioactives [67]. 

Overall, gelation- and extrusion-based techniques represent versatile and food-grade approaches for the encapsulation of bioactive compounds recovered from food-industry by-products, offering good protection against environmental degradation and the possibility of designing targeted delivery systems for functional food applications.

### 4.2. Drying Techniques for the Stabilization of Encapsulated Systems

Drying techniques play an important role in the stabilization and preservation of encapsulated bioactive compounds intended for food applications [47,80]. These technologies are among the most widely used approaches for improving the storage stability, handling properties, and incorporation of bioactive compounds recovered from food-industry by-products into functional food formulations [51,59,60].

#### 4.2.1. Spray Drying

Spray drying is the most widely used encapsulation and drying technique in the food industry because it is cost-effective, easily scalable, and suitable for the production of stable powders with extended shelf life [46,47,53,81]. Although some authors consider spray drying primarily as a dehydration process rather than a true microencapsulation technique, it remains one of the most frequently applied approaches for stabilizing bioactive compounds and facilitating their incorporation into food systems [46,47].

The method has been extensively used for the valorization of food-industry by-products rich in phytochemicals. Phenolic compound-rich extracts obtained from grape pomace, pomegranate peel, citrus peels, and berry processing residues have been successfully converted into stable powders using carriers such as maltodextrin, gum arabic, proteins, and modified starches [35,51,59,82,83,84,85]. Spray drying significantly improves the storage stability of phenolic compounds, reduces oxidation, and enhances the handling and incorporation of extracts into beverages, dairy products, and bakery formulations [46,51].

The technique has also been successfully applied to the encapsulation of anthocyanins and carotenoids recovered from fruit and vegetable processing by-products. For example, anthocyanin-rich extracts from black chokeberry pomace and carotenoid-rich fractions obtained from fruit peels and tomato pomace have been successfully converted into spray-dried powders [85,86,87]. Encapsulation by spray drying can enhance the stability of sensitive phytochemicals during storage and reduce their degradation associated with environmental factors such as light, oxygen, and temperature, although the extent of protection strongly depends on the carrier material and storage conditions [87,88,89].

Beyond phytochemical encapsulation, spray drying is also widely employed for the protection of probiotic microorganisms. Ohja et al. [90] encapsulated *Lactiplantibacillus plantarum* CRD7 in submicron pullulan-based particles produced by spray drying and demonstrated that the incorporation of prebiotic and thermal protective agents improved probiotic survival during processing. This example illustrates how appropriate carrier composition can mitigate the thermal and dehydration stresses associated with spray drying and extend the applicability of this technology to sensitive biological ingredients.

Despite its numerous advantages, spray drying may not be suitable for highly thermolabile or volatile compounds because exposure to elevated temperatures can result in partial thermal degradation and lower encapsulation efficiency [47,53]. Nevertheless, owing to its simplicity, low operational cost, and industrial feasibility, spray drying remains one of the most important and commercially viable technologies for the stabilization and sustainable valorization of bioactive compounds recovered from agro-industrial residues [51].

#### 4.2.2. Freeze Drying

Freeze drying (lyophilization) is widely applied for the stabilization of thermolabile bioactive compounds because it minimizes thermal degradation and preserves the structural integrity of sensitive molecules [47,53,88]. Although the process is characterized by high operational costs and long processing times, it is considered one of the most effective methods for retaining the biological activity of encapsulated compounds [47]. During freezing, water crystallizes as ice; during primary drying, ice is subsequently removed by sublimation under reduced pressure, thereby limiting thermal stress [46].

The technique has been successfully employed for the stabilization of phenolic compound-rich extracts recovered from food-industry by-products, including grape pomace, berry residues, and fruit peels [59,67,81]. Several studies have demonstrated that freeze-dried microcapsules exhibit higher retention of phenolic compounds and antioxidant activity than powders produced by conventional thermal drying methods [67]. For instance, freeze-drying of onion skin phenolic compounds yielded substantially higher encapsulation efficiencies (66.6–78.0%) compared to spray-dried systems (10.1–14.1%), preserving up to 98.8% of flavonoids after storage [91]. In particular, freeze-drying effectively preserves anthocyanins and other oxidation-sensitive phytochemicals within polymer matrices, such as gum arabic, which shield hydrophilic bioactive compounds, including chlorogenic and quinic acids, from oxidative degradation and promote excellent biocompatibility [60].

Freeze drying is also frequently used as a post-encapsulation stabilization step for hydrogel particles produced by ionic gelation, complex coacervation, or extrusion techniques [46,67]. The sublimation of water immobilizes the core within an amorphous glassy matrix, which raises the glass transition temperature (Tg) of the powder (typically around 50–52 °C for pectin or maltodextrin systems), thereby preventing structural collapse, reducing hygroscopicity, and facilitating their incorporation into powdered functional foods and nutraceutical formulations [59,60]. Successful applications include freeze-dried calcium alginate beads containing phenolic-rich extracts recovered from spent sour cherry pomace [92] and sage-loaded coacervates based on whey protein and gum arabic [93].

Despite its excellent protective capacity, the industrial application of freeze drying remains limited by its high energy requirements, prolonged processing cycles, and relatively low economic efficiency [47,53]. Nevertheless, the method remains particularly attractive for high-value phytochemicals recovered from agro-industrial residues, where maximizing bioactive retention and maintaining structural and chemical integrity are of greater importance than minimizing processing costs [47,60].

#### 4.2.3. Fluidized-Bed Drying

Fluidized-bed drying is primarily employed as a secondary drying and coating technique to improve the stability, flowability, and storage properties of encapsulated powders. Unlike spray drying and freeze drying, it is less frequently used as a standalone encapsulation method, but it offers the advantage of simultaneously drying and coating particles, thereby providing an additional protective barrier for sensitive bioactive compounds [47,80].

The technique has been investigated for the stabilization of phenolic extracts and essential oils, where the application of additional coating layers improved oxidative stability and prolonged the shelf life of encapsulated ingredients. Fluidized-bed processing has also been used to produce multilayer particles intended for incorporation into functional foods, particularly when enhanced protection during storage and processing is required [53,89].

Because of its ability to combine drying and coating operations, fluidized-bed technology represents a useful complementary approach for the production of stable functional ingredients from food-industry by-products, although its application remains less widespread than that of spray drying and freeze drying [51].

### 4.3. Particle Formation Techniques

Particle formation techniques represent promising engineering approaches for improving the physicochemical properties, stability, and delivery of poorly water-soluble phytochemicals recovered from food-industry by-products [54,94]. By enabling the controlled production of microparticles and nanoparticles with tailored size, surface properties, and internal structure, these technologies can help overcome important limitations of by-product-derived bioactive extracts, including poor aqueous dispersibility, chemical instability, and limited intestinal bioaccessibility [53,54]. Reducing particle size to the submicron or nanometer range increases the surface-to-volume ratio, which can enhance dissolution rates and improve the aqueous dispersibility of poorly water-soluble bioactive compounds. This is particularly relevant for hydrophobic phytochemicals such as carotenoids, phytosterols, and lipophilic flavonoids. Depending on the formulation and carrier system, particle engineering may also improve gastrointestinal bioaccessibility and, in some cases, bioavailability, thereby facilitating the incorporation of these compounds into functional food products [46,57,94].

#### 4.3.1. Antisolvent Precipitation

Antisolvent precipitation is a mild, low-temperature approach for improving the technological functionality, aqueous dispersibility, and bioaccessibility of poorly water-soluble bioactive compounds [54,94]. The process is based on rapid supersaturation induced by controlled solvent–antisolvent mixing, which promotes nucleation and enables the bottom-up formation of submicron or nanoscale particles. The resulting increase in surface-to-volume ratio can enhance dissolution rates and improve the dispersibility of hydrophobic compounds under relatively mild processing conditions [54,94]. Owing to its relatively simple operation and limited thermal exposure, the technique is particularly attractive for thermolabile and oxidation-sensitive phytochemicals intended for food applications [46].

The method has been extensively investigated for the formulation of hydrophobic phytochemicals, including phenolic compounds, flavonoids, and carotenoids. Curcumin and resveratrol are frequently used as model compounds because of their poor water solubility and limited gastrointestinal bioaccessibility, and their formulation as submicron or nanoscale particles can improve their dispersibility and dissolution behavior [94,95]. Similar approaches may also be useful for hydrophobic bioactive compounds recovered from agro-industrial by-products, including carotenoid- and polyphenol-rich fractions, whose incorporation into aqueous food systems is often limited by poor water solubility and chemical instability [51,59].

The resulting colloidal dispersions may facilitate the incorporation of lipophilic bioactive compounds into beverages, dairy products, and other aqueous food formulations [54]. However, industrial application remains constrained by several technological challenges, including the removal of residual organic solvents, precise control of mixing conditions to regulate nucleation and particle growth, and the use of suitable food-grade stabilizers to minimize particle aggregation and recrystallization during storage [46,47]. Overall, antisolvent precipitation represents a promising strategy for improving the technological functionality and bioaccessibility of poorly soluble phytochemicals and may support the development of value-added functional ingredients from food-industry by-products.

#### 4.3.2. Supercritical Fluid Encapsulation

Supercritical fluid technologies have gained considerable attention as sustainable alternatives to conventional solvent-based encapsulation methods because they enable the production of microparticles and nanoparticles under relatively mild processing conditions while minimizing the use of organic solvents [96,97,98]. Among the available supercritical fluids, supercritical carbon dioxide (scCO_2_) is the most widely used in food-related applications owing to its low critical temperature (31.1 °C), moderate critical pressure (7.38 MPa), non-flammability, and easy removal from the final product by depressurization. Depending on the process configuration, scCO_2_ can act as a solvent, antisolvent, or atomization aid, enabling the production of microparticles and nanoparticles with controlled size and morphology [57,96,98].

These technologies have been successfully applied to the encapsulation of poorly soluble and oxidation-sensitive bioactive compounds, including phenolic compounds, carotenoids, essential oils, and lipid fractions [57,98,99]. Their main advantages include limited thermal exposure and the possibility of tailoring particle characteristics through process parameters such as pressure, temperature, flow rate, and carrier-to-core ratio. Because pure scCO_2_ is relatively non-polar, food-grade co-solvents such as ethanol may be used to facilitate the processing of more polar bioactive compounds or complex plant extracts [100].

Supercritical technologies are also particularly relevant to the valorization of agro-industrial by-products. Supercritical-assisted atomization (SAA) has been used to encapsulate bioactive compounds recovered from olive pomace and apple pomace, producing stabilized particulate ingredients with potential food applications [101,102]. Supercritical extraction may also be integrated with subsequent particle formation or encapsulation within cascade biorefinery systems, for example for the recovery and stabilization of carotenoids and phenolic compounds from fruit and vegetable processing residues [57,100,103]. This integration supports the conversion of food-industry by-products into high-value functional ingredients while reducing reliance on conventional organic solvents.

### 4.4. Emulsion-Based Encapsulation Systems

Emulsion-based delivery systems represent one of the most versatile and extensively investigated approaches for the encapsulation and delivery of food bioactive compounds [47,48]. Their widespread use is largely due to their ability to improve the aqueous dispersibility, chemical stability, and gastrointestinal bioaccessibility of poorly soluble bioactive compounds while remaining compatible with a wide range of food matrices [48]. In addition to serving as direct delivery systems, emulsions can act as precursor structures for more complex encapsulation formats, including spray-dried particles, multilayer microcapsules, and biopolymer-based gel carriers [59,104]. Consequently, emulsion technologies are increasingly investigated for the stabilization and incorporation of sensitive bioactive compounds recovered from agro-industrial by-products into functional food formulations. Depending on droplet size, internal structure, and stabilization mechanism, these systems include conventional emulsions, nanoemulsions, Pickering emulsions, and multiple emulsions, including double emulsions, which are discussed in the following subsections.

#### 4.4.1. Conventional Emulsions and Nanoemulsions

Conventional emulsions and nanoemulsions are among the most versatile delivery systems for food bioactive compounds owing to their ability to improve the dispersibility, stability, and bioaccessibility of poorly water-soluble ingredients [48,105]. Nanoemulsions, typically characterized by droplet diameters below 200 nm, exhibit improved kinetic stability and often provide enhanced gastrointestinal bioaccessibility of encapsulated compounds compared with conventional emulsions [105].

Emulsion-based systems have been extensively investigated for the encapsulation of phenolic compounds, carotenoids, essential oils, and lipid-soluble vitamins recovered from food-industry by-products. Phenolic extracts obtained from grape pomace and olive processing residues have been successfully incorporated into nanoemulsions, resulting in improved oxidative stability, enhanced antioxidant activity, and increased bioaccessibility of encapsulated compounds [57,106]. Likewise, carotenoids and essential oils recovered from citrus processing by-products have been formulated into nanoemulsions with improved dispersibility and protection against environmental degradation.

Several studies have demonstrated that emulsion-based systems facilitate the incorporation of recovered phytochemicals into food matrices that would otherwise be unsuitable because of the poor water solubility or instability of the active compounds. Nanoemulsions containing phenolic compounds and carotenoids have been successfully incorporated into beverages, dairy products, and functional ingredients while maintaining their antioxidant activity and improving their bioaccessibility [48].

Importantly, emulsion-based systems should not be viewed solely as carriers for lipophilic compounds, as they also serve as versatile precursor systems for subsequent encapsulation processes, including spray drying, freeze drying, coacervation, and layer-by-layer deposition.

Their flexibility, scalability, and ability to improve the stability and gastrointestinal bioaccessibility of bioactive compounds make conventional emulsions and nanoemulsions promising delivery platforms for the valorization of food-industry by-products and the development of next-generation functional foods. Nevertheless, long-term physical stability and susceptibility to environmental stresses remain important challenges that require further investigation.

#### 4.4.2. Pickering Emulsions

Pickering emulsions are emulsion systems stabilized by solid particles adsorbed at the oil–water interface rather than by conventional surfactants. In recent years, these systems have attracted considerable attention because of their exceptional physical stability, reduced dependence on synthetic emulsifiers, and compatibility with clean-label food formulations [107,108,109].

A wide range of food-grade particles has been investigated as Pickering stabilizers, including proteins, polysaccharides, cellulose nanocrystals, starch particles, and dietary fibers [108,110,111]. Particularly promising are particles derived from agro-industrial by-products, which may simultaneously serve as stabilizers and value-added ingredients, further supporting circular economy strategies and sustainable food production [111,112]. Unlike conventional emulsions, Pickering systems offer the unique possibility of employing particles recovered from food-processing residues as both stabilizing agents and functional ingredients. Pickering emulsions have been successfully employed for the encapsulation and protection of phenolic compounds, carotenoids, essential oils, and other oxidation-sensitive bioactive compounds. Several studies have demonstrated improved chemical stability, controlled release, and enhanced bioaccessibility of encapsulated compounds compared with conventional emulsions [48,108,110].

The application of Pickering emulsions for the valorization of food-industry by-products has expanded considerably in recent years. Cellulose particles recovered from fruit and vegetable residues, protein nanoparticles obtained from oilseed processing by-products, and insoluble dietary fibers derived from pomace have all been investigated as natural stabilizers for emulsion-based delivery systems [110,111,112]. Furthermore, Pickering emulsions have been successfully used to encapsulate phenolic extracts recovered from grape pomace and citrus processing residues, resulting in improved oxidative stability and facilitating their incorporation into functional food products [48,108,110].

Because of their excellent stability, clean-label potential, and compatibility with food-grade particles obtained from agro-industrial residues, Pickering emulsions represent one of the most promising emerging platforms for the stabilization and delivery of bioactive compounds recovered from food-industry by-products [110,111]. Nevertheless, further studies are required to optimize particle production, improve process scalability, and better understand the gastrointestinal fate of these systems.

#### 4.4.3. Double Emulsions and Emulsion-Based Coacervation Systems

Multiple emulsions, particularly water-in-oil-in-water (W/O/W) double emulsions, provide additional opportunities for the encapsulation of hydrophilic bioactive compounds within the internal aqueous phase while simultaneously allowing lipophilic components to be incorporated into the oil phase. Their compartmentalized structure may therefore facilitate the co-encapsulation of compounds with different solubility characteristics and provide enhanced protection of particularly sensitive functional ingredients. 

Double emulsions may also serve as templates for subsequent encapsulation processes such as complex coacervation, in which electrostatic interactions between oppositely charged biopolymers, typically proteins and polysaccharides, promote the formation of protective interfacial or capsule structures around the dispersed droplets [113,114]. Such hybrid systems have been investigated for the stabilization of anthocyanins, phenolic extracts, probiotics, and other sensitive functional ingredients, including compounds originating from plant-derived materials and food-processing by-products [104,114,115].

The combination of multiple-emulsion systems with biopolymer-based encapsulation may improve protection against processing and gastrointestinal stresses while also enabling modulation of bioactive release. However, the structural complexity of double emulsions, their sensitivity to formulation and processing conditions, and the additional technological steps required for their production remain important limitations for large-scale food applications.

### 4.5. Complex Coacervation

Complex coacervation is an encapsulation technique based on the electrostatic interaction between oppositely charged biopolymers, typically proteins and polysaccharides, resulting in the formation of a polymer-rich phase capable of entrapping active compounds [47,113]. Owing to its mild processing conditions and high encapsulation efficiency, complex coacervation has been widely investigated for the protection of oxidation-sensitive bioactive compounds intended for food applications. This technique has been successfully applied to the encapsulation of phenolic compounds, anthocyanins, essential oils, and lipid-soluble bioactives. In particular, coacervate systems have demonstrated excellent retention of sensitive compounds and the ability to provide controlled release during gastrointestinal digestion [57,114,116].

The application of complex coacervation to bioactive compounds recovered from food-industry by-products has expanded considerably in recent years. For example, grape by-product polyphenols have been successfully encapsulated using casein–pectin complex coacervation combined with spray drying, which substantially reduced phytochemical degradation during processing [117]. Pomegranate peel extract has likewise been encapsulated in *Prunus armeniaca* gum exudate–chitosan coacervate microparticles, providing sustained release and effective protection of phenolic compounds under simulated gastrointestinal conditions [118]. Anthocyanin-rich plant extracts have also been successfully stabilized using protein–polysaccharide complex coacervation systems, with improvements in anthocyanin retention, physicochemical stability, and protection during storage [119,120].

Gelatin, whey proteins, gum arabic, pectin, alginate, and chitosan are among the most frequently employed food-grade materials because of their excellent film-forming properties and compatibility with food systems [113,114].

Because of its high encapsulation efficiency, ability to protect sensitive compounds, and potential to provide controlled release, complex coacervation represents a promising strategy for the stabilization and delivery of bioactive compounds recovered from food-industry by-products. Furthermore, the use of food-grade biopolymers and relatively mild processing conditions makes this approach particularly attractive for the development of functional food ingredients and clean-label formulations. Nevertheless, challenges associated with process scalability and the stability of coacervates under varying environmental conditions still require further investigation.

### 4.6. Lipid-Based Delivery Systems

Lipid-based delivery systems have emerged as highly effective carriers for food bioactive compounds owing to their ability to improve the solubility, stability, and gastrointestinal bioaccessibility and, in some cases, bioavailability of poorly water-soluble and oxidation-sensitive ingredients. These systems are particularly attractive for the encapsulation of phenolic compounds, carotenoids, essential oils, and lipid-soluble vitamins because they protect encapsulated compounds against environmental degradation and enhance their gastrointestinal bioaccessibility [54,95]. Recent studies have also highlighted their potential for incorporating bioactive compounds recovered from food-industry by-products into functional food matrices, including beverages, dairy products, bakery products, and edible coatings.

#### 4.6.1. Liposomes

Liposomes are phospholipid vesicles capable of encapsulating both hydrophilic and lipophilic compounds, making them highly versatile delivery systems for food applications [47,121]. Owing to their biocompatibility and ability to enhance gastrointestinal bioaccessibility, liposomes have been widely investigated as food delivery systems. 

More recently, polyphenol-rich extracts recovered from red grape pomace have been successfully incorporated into nanoliposomal systems, demonstrating the applicability of phospholipid-based carriers to the valorization of winery by-products [122]. Such systems may improve the stability and delivery of sensitive phytochemicals and facilitate their incorporation into functional food formulations. 

Despite their advantages, the industrial application of liposomes remains limited by their relatively high production costs and susceptibility to oxidation and leakage during storage. Therefore, current research focuses on developing hybrid and multilayer liposomal systems with improved stability and functionality.

#### 4.6.2. Solid Lipid Nanoparticles (SLNs)

Solid lipid nanoparticles (SLNs) are colloidal carriers composed of lipids that remain solid at both room and body temperatures. Owing to their ability to protect sensitive compounds against oxidation and improve the dispersibility and gastrointestinal bioaccessibility of poorly water-soluble ingredients, SLNs have attracted considerable interest as delivery systems for food bioactive compounds [94,95].

Recent studies have demonstrated the successful application of SLNs for the encapsulation of phenolic compounds, carotenoids, essential oils, and other phytochemicals relevant to the valorization of food-industry by-products. Grape seed-derived proanthocyanidins have been successfully incorporated into SLNs, demonstrating effective protection and preservation of antioxidant and anti-inflammatory activity in vitro [123]. Similarly, olive leaf extract rich in oleuropein was encapsulated in SLNs with an encapsulation efficiency of approximately 80%, producing a stable formulation with sustained release and enhanced radical-scavenging activity [124]. Tomato by-product-derived steroidal glycoalkaloids, including α-tomatine and tomatidine, have also been successfully formulated in SLNs, further demonstrating the potential of lipid nanoparticles for the valorization of processing residues [125]. In addition, essential oils obtained from citrus peels have been incorporated into SLNs, demonstrating effective encapsulation and controlled release of volatile compounds [126]. Carotenoid encapsulation in SLNs has also been demonstrated for lycopene, with high encapsulation efficiency and good physicochemical stability; however, direct studies using carotenoids specifically recovered from food-processing by-products remain comparatively limited [127].

Overall, SLNs provide a promising platform for the stabilization and delivery of lipophilic and oxidation-sensitive bioactive compounds relevant to functional food development. However, their highly ordered crystalline lipid structure may restrict loading capacity and promote the expulsion of encapsulated compounds during storage as the lipid matrix undergoes structural reorganization [94,95].

#### 4.6.3. Nanostructured Lipid Carriers (NLCs)

Nanostructured lipid carriers (NLCs) represent the second generation of lipid nanoparticles and consist of a mixture of solid and liquid lipids, resulting in a less ordered internal structure and improved loading capacity compared with SLNs [94,95]. Owing to their high encapsulation efficiency and ability to protect sensitive compounds against environmental degradation, NLCs have attracted considerable interest as delivery systems for poorly water-soluble food bioactive compounds. While conventional NLCs rely on optimizing the ratios of solid and liquid lipids at the nanoscale, recent trends point to the potential of creating microscopic “oleogel-in-oleogel” structures, where the precise architecture of the lipid matrix enables direct control over lipolysis in the gastrointestinal tract [128].

NLCs have been extensively investigated for the encapsulation of phenolic compounds, carotenoids, essential oils, and other phytochemicals relevant to the valorization of food-industry by-products. Lycopene has been successfully incorporated into NLCs, resulting in improved chemical stability, aqueous dispersibility, and gastrointestinal bioaccessibility [129,130]. More recently, carotenoids recovered directly from carrot peels, including α-carotene, β-carotene, lycopene, and lutein, were successfully incorporated into NLCs, demonstrating the potential of these systems for coupling bioactive recovery from processing residues with subsequent nanoencapsulation [131].

Similarly, NLC-based formulations containing phenolic compounds and phenolic-rich plant extracts have demonstrated improved stability, antioxidant activity, and sustained or controlled release compared with non-encapsulated compounds [132,133,134,135].

However, improvements in dispersibility, stability, or in vitro gastrointestinal bioaccessibility should not be interpreted as evidence of demonstrated health benefits. Before industrial application in foods, nano-delivery systems require confirmation of their food-grade suitability, appropriate toxicological assessment, evaluation of their gastrointestinal fate, and compliance with applicable regulatory requirements.

### 4.7. Cyclodextrin Inclusion Complexes

Cyclodextrins have been widely applied for the stabilization of phenolic compounds and volatile phytochemicals because they improve solubility, protect against oxidation, and reduce undesirable sensory attributes [136,137]. Their application has recently expanded toward the valorization of food-industry by-products, particularly as carriers for bioactive compounds recovered from fruit and vegetable processing residues.

β-cyclodextrin has been employed for the simultaneous extraction and encapsulation of phenolic compounds from blackthorn fruits and other fruit by-products, resulting in enhanced antioxidant activity and improved storage stability of the recovered compounds [138]. Aqueous β-cyclodextrin has also been successfully employed for the recovery of stilbenes, flavonols, and flavan-3-ols from grape pomace, illustrating the potential of cyclodextrins to enhance the extraction of phenolic compounds while simultaneously promoting their molecular complexation [139]. In addition, cyclodextrin inclusion complexes have been successfully used to stabilize citrus essential oils and olive phenolics, including hydroxytyrosol and oleuropein, reducing their susceptibility to oxidation and facilitating their incorporation into functional food formulations [136,140].

The ability of cyclodextrins to improve the physicochemical properties of phytochemicals while simultaneously enhancing their stability and applicability in food systems highlights their potential as food-grade carriers for the valorization of food-industry by-products. Nevertheless, further studies are required to evaluate their performance in complex food matrices and to assess their industrial scalability.

### 4.8. Layer-by-Layer Systems

Layer-by-layer (LbL) technology has recently attracted attention as an advanced strategy for designing multilayer emulsions and lipid-based delivery systems with improved stability and tailored release characteristics. This electrostatic deposition approach offers precise control over the coating thickness, permeability, and mechanical strength of the carrier shell, making it a highly tunable platform for the protection of sensitive food ingredients [141]. In food applications, LbL systems have been primarily employed to encapsulate phenolic compounds, carotenoids, and essential oils, thereby improving their protection against oxidation and gastrointestinal degradation [142,143].

Recent studies have demonstrated the successful incorporation of extracts rich in phenolic compounds recovered from fruit-processing by-products into multilayer emulsion systems, resulting in improved storage stability and enhanced bioaccessibility of encapsulated compounds [143,144].

Although industrial applications remain limited, the ability of LbL systems to engineer multilayer structures with customized release profiles makes this approach a promising emerging technology for the valorization of food-industry by-products and the development of next-generation functional foods [142,144]. Table 1 provides an overview of the main encapsulation approaches, highlighting their principles, advantages, limitations, and potential applications in functional food development.

From an application-oriented perspective, the most promising encapsulation approach depends on the intended product and processing constraints. Spray drying is a strong candidate for large-volume powdered ingredients because of its established industrial use and scalability, provided that the formulation tolerates the drying conditions [47,81]. Freeze drying may be justified for high-value, thermolabile extracts when improved retention offsets its greater processing costs; however, superior laboratory performance alone does not establish industrial feasibility [99]. Emulsion-based and lipid-based systems offer opportunities for incorporating poorly water-soluble compounds into aqueous foods, but their suitability depends on storage stability, ingredient compatibility, and food-grade formulation requirements. Thus, technological sophistication should not be treated as evidence of practical superiority: the preferred system is the one that provides adequate protection and delivery at a feasible cost and production scale.

**Figure 1:**
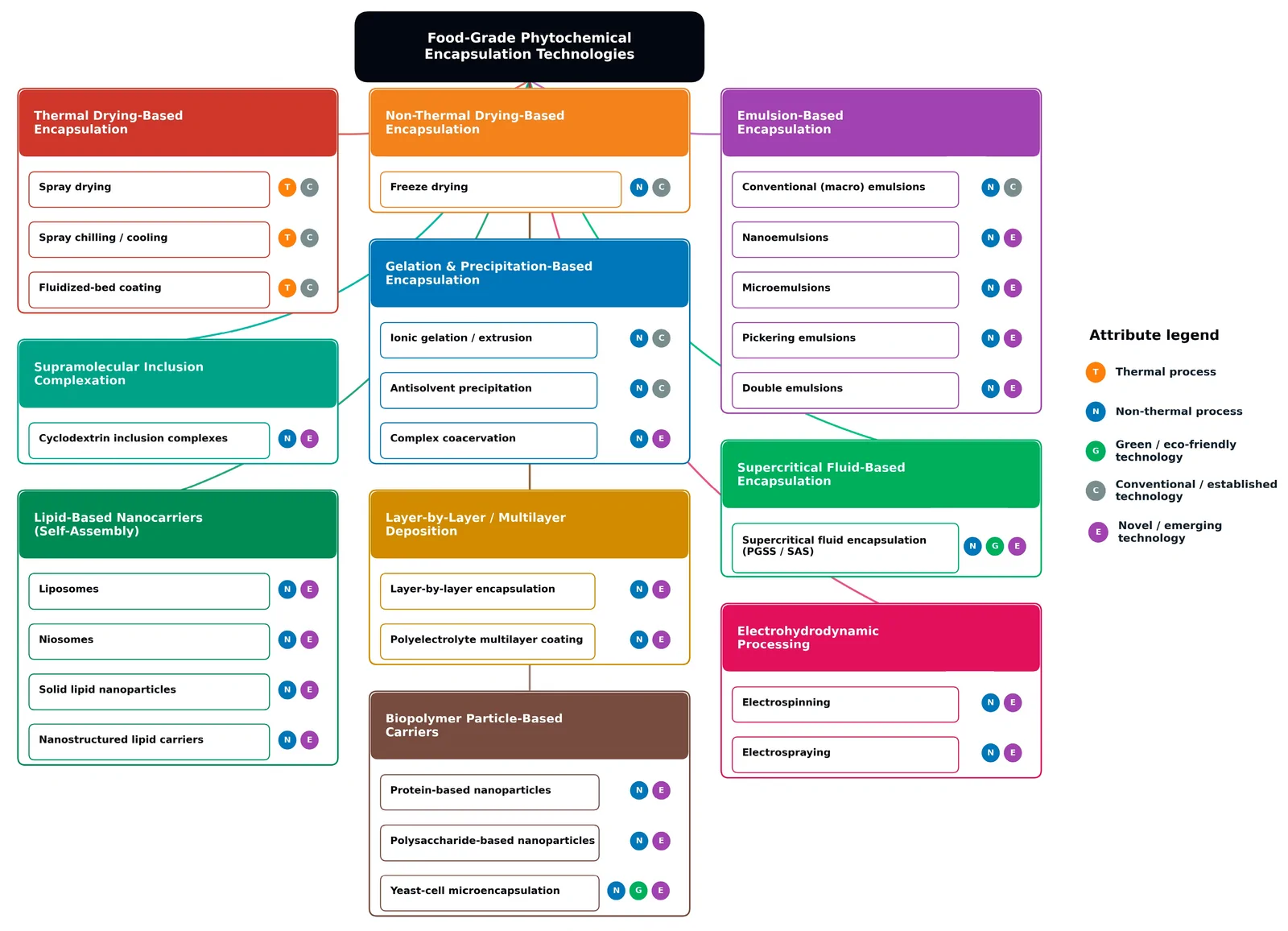
Schematic classification of the encapsulation technologies discussed in this review according to their underlying mechanisms, with individual methods identified as thermal or non-thermal and as conventional/established, green/eco-friendly, or novel/emerging technologies for stabilizing bioactive compounds recovered from food-industry by-products.

For clarity, the encapsulation technologies discussed in this review can be broadly classified into conventional thermal and non-thermal methods, green technologies, and novel and emerging delivery systems, as illustrated in Figure 1.

## 5. Extraction of Phytochemicals from Food-Processing By-Products

Agro-industrial by-products such as peels, pomace, seeds, and residual fractions constitute highly concentrated matrices of bioactive phytochemicals and are increasingly recognized as strategic secondary raw materials for high-value compound recovery [50,51]. In many plant species, valuable phenolics, carotenoids, and anthocyanins are preferentially localized in outer tissues (such as the cuticle or epidermal layer) involved in defense against environmental and biotic stress; consequently, these by-products often contain concentrations of bioactive compounds comparable to, or even exceeding, those found in the edible plant parts typically consumed [50]. Their systematic valorization should therefore be understood not merely as a waste-management strategy, but as a scientifically substantiated approach to functional ingredient innovation within circular bioeconomy frameworks [51,59]. As summarized in Table 2, both polar and non-polar phytochemicals can be efficiently recovered from these matrices when advanced green extraction technologies and process parameters are carefully optimized. However, extraction yield alone is insufficient as a performance indicator; it must be interpreted in conjunction with extraction selectivity, molecular stability, downstream processing demands, safety standards, and overall techno-economic feasibility [51,59]. In this context, extraction yield refers to the amount of extract obtained relative to the starting material, whereas recovery refers specifically to the proportion of a target compound recovered from the original matrix; these parameters should therefore not be interpreted interchangeably.

The efficiency of phenolic compound recovery from agricultural residues strongly depends on the extraction technology employed. Enzyme-assisted extraction (EAE) can enhance the release of intracellular and cell-wall-associated compounds through the hydrolysis of structural polysaccharides. The importance of matching enzyme composition to the plant matrix is illustrated by Mazzocchi et al. [145], who developed a tailored enzymatic system composed of cellulase, xylanase, and polygalacturonase for the recovery of chlorophyll from unsold spinach. Although this study targeted chlorophyll rather than phenolic compounds, it illustrates a matrix-specific approach to enzyme selection. For phenolic recovery, the use of pectinolytic and cellulolytic enzymes can enhance extraction efficiency by disrupting plant cell-wall structures and facilitating the release of matrix-associated phenolic compounds [146]. However, the broader industrial application of enzyme-assisted extraction may be limited by processing costs, enzyme stability, and regulatory requirements associated with food-grade applications [51]. Enzymatic treatment may also modify the phenolic composition of the resulting extracts or juices, with potential implications for their sensory properties [147,148]. Moreover, extensive degradation of cell-wall polysaccharides by cellulolytic enzymes may affect the properties and subsequent recovery of structural components, including pectin [149]. 

Extraction should therefore be considered within the overall fractionation strategy rather than optimized solely for a single target compound. To improve the comprehensive utilization of this biomass within a circular bioeconomy, recent technological approaches increasingly focus on cascade biorefinery concepts that enable the sequential recovery of multiple valuable fractions, including phenolic compounds and pectin [51]. For example, combined recovery of pectin and phenolic compounds from apple pomace illustrates the potential for obtaining complementary products from the same raw material [73]. In parallel, emerging green extraction technologies, such as subcritical water extraction (SWE), offer promising opportunities for fractionating agricultural by-products into bioactive-rich extracts and fiber-rich residual fractions while reducing reliance on conventional organic solvents [59]. These approaches highlight the importance of evaluating extraction performance in relation to both the recovered phytochemicals and the potential uses of the remaining biomass. Table 2 summarizes representative extraction technologies and operating conditions, providing a basis for comparing recovery, selectivity, and processing requirements.

**Table 2:** Summary of advanced extraction technologies applied to food processing by-products for the recovery of bioactive compounds.

Source	Target Compounds	Method	Extraction Conditions	Key Findings	**Ref.**
Carrot juice processing waste	Carotenoids	MAE	Solvent: flaxseed oil; oil-to-waste ratio: 8.06:1 (g/g); time: 9.39 min; microwave power: 165 W	Carotenoid recovery of 77.48%; the resulting carotenoid-enriched flaxseed oil also exhibited high phenolic content and antioxidant activity.	[150]
Lemon peel waste	Lemon essential oil (LEO); lemon pigments (LP)	Sequential MAHD + MAE	LEO: microwave-assisted hydrodistillation (MAHD) using a Clevenger apparatus. LP: 80% EtOH; solid-to-liquid ratio: 1:10; 80 °C; 50 min	Extraction yields of approximately 2 wt.% for LEO and 6 wt.% for LP. Major LEO components: limonene (65.1%), β-pinene (14.5%), and γ-terpinene (9.7%). Major LP compounds: eriocitrin (4.7%), diosmin (7.4%), and hesperidin (2.7%).	[151]
Pomegranate peels	Phenolic compounds	VMAAE	Conditions optimized for TPC: water/peel ratio: 39.92%; 61.48 °C; 10 min; 3797.24 W. Conditions optimized for antioxidant activity: water/peel ratio: 38.2%; 79.16 °C; 12.17 min; 3576.47 W	Optimized VMAAE yielded extracts with high TPC (5.542 mg GAE/g fresh peel/min) and strong DPPH radical-scavenging activity.	[152]
Tomato peel by-product	Lycopene; β-carotene	Supercritical CO_2_ extraction	CO_2_; temperature: 50–80 °C; pressure: 300–500 bar; extraction time: 105 min; CO_2_ flow rate: 3–6 g/min	Relative extraction yields ranged from 32.02 to 60.85% for lycopene and from 28.38 to 58.8% for β-carotene. The resulting oleoresins also showed substantial antioxidant activity.	[153]
Apple pomace and peel	Phenolic compounds; triterpenic acids	UAE	68% EtOH; solid-to-liquid ratio: 1:75; extraction time: 5.1 min; ultrasound amplitude: 90%	Whole apple pomace contained approximately 1300 µg/g flavonols, 1200 µg/g dihydrochalcones, and 4200 µg/g ursolic acid (dry matter), increasing in apple peel to approximately 2500, 1400, and 8500 µg/g, respectively.	[154]
Pomegranate seeds	Oil; bioactive compounds, particularly punicic acid	UAE	Solvent: *n*-hexane; optimal liquid-to-solid ratio: 19 mL/g; time: 12 min; amplitude: 46%	UAE provided an oil yield of 12.67% and the highest oxidative stability (τmax = 5.44 min). It produced a higher extraction yield than cold pressing and Soxhlet extraction, although total polyphenol content and antioxidant activity were slightly lower than in cold-pressed oil.	[155]
Black chokeberry pomace	Polyphenols; anthocyanins	PLE, MAE, UAE	Solvent: 50% aqueous EtOH + 1% formic acid. PLE: 100–150 °C, 5–10 min, solid-to-solvent ratio 1:40–1:80, 10.34 MPa. MAE: 40–80 °C, 5–10 min, 400 W, ratio 1:40–1:80. UAE: <30 °C, 5–10 min, amplitude 50–100%, ratio 1:40–1:80.	TP (mg GAE/g dm): 101.3 (PLE), 94.8 (MAE), and 79.9 (UAE); TMA (mg C3GE/g dm): 19.3, 24.0, and 23.2, respectively. MAE provided a favorable balance between polyphenol and anthocyanin recovery and high antioxidant capacity.	[156]

Abbreviations: MAE—microwave-assisted extraction; MAHD—microwave-assisted hydrodistillation; VMAAE—vacuum microwave-assisted aqueous extraction; UAE—ultrasound-assisted extraction; PLE—pressurized liquid extraction; MAC—maceration; DES—deep eutectic solvent; CO_2_—carbon dioxide; EtOH—ethanol; LEO—lemon essential oil; LP—lemon pigments; TPC—total phenolic content; TP—total polyphenols; TMA—total monomeric anthocyanins; GAE—gallic acid equivalents; C3GE—cyanidin-3-glucoside equivalents; DPPH—2,2-diphenyl-1-picrylhydrazyl radical scavenging assay; τmax—maximum oxidation induction time; RSD—relative standard deviation; dm—dry matter; *w/v—*weight per volume; *v/v—*volume per volume.

A fundamental determinant of extraction performance is the alignment between compound polarity, matrix structure, and extraction technology. This relationship is particularly evident in carotenoid-rich biowaste. Microwave-assisted extraction (MAE) enabled carotenoid recovery of 77.48% from carrot by-products [150], demonstrating the effectiveness of microwave-induced volumetric heating, which promotes rapid internal temperature rise, cell wall disruption, and enhanced mass transfer of lipophilic compounds. Similarly, supercritical CO_2_ extraction of tomato peels yielded relative lycopene extraction yields of up to 60.85% under optimized pressure and temperature conditions [153], confirming the suitability of supercritical fluids for non-polar, oxidation-sensitive bioactives where solvent residues must be minimized. The potential of this approach has also been demonstrated at the biorefinery level. Scaglia et al. [157] integrated supercritical CO_2_ extraction of tomato pomace into a cascade valorization process aimed at obtaining a high-value lycopene-rich fraction while simultaneously generating bioenergy and nutrient-rich digestate from the remaining biomass. This integrated strategy illustrates how selective recovery of lipophilic bioactives can be combined with the valorization of residual fractions, thereby improving the overall resource efficiency of food-processing by-product utilization. These examples illustrate that energy-assisted and supercritical techniques are particularly appropriate for lipophilic compounds embedded in complex plant matrices [158]. However, attention should also be paid to the selectivity of extraction processes, solvent consumption, and the need for solvent removal, recovery, and purification, as well as to the additional energy demand associated with these technologies. Technological suitability must therefore be balanced against industrial scalability and energy demand. MAE offers rapid processing and reduced solvent consumption, yet its scale-up is limited by microwave penetration depth and challenges in achieving homogeneous heating in larger processing volumes. Supercritical CO_2_ extraction provides organic-solvent-free recovery and high selectivity; however, high capital investment and substantial compression energy requirements generally restrict its application to high-value specialty ingredients. Consequently, its economic viability is greater in the production of premium nutraceuticals or concentrated carotenoid preparations than in large-volume antioxidant ingredients intended for cost-sensitive functional or fortified food markets. 

It should be emphasized that improved extraction yield, reduced solvent consumption, or shorter processing time do not, by themselves, demonstrate environmental sustainability. These are important green-processing attributes, but the overall environmental performance of an extraction process also depends on energy demand, solvent production and recovery, water use, downstream purification and drying, and process scale. This is particularly relevant for MAE and supercritical CO_2_ extraction, where reduced solvent use or improved selectivity may be accompanied by substantial electricity or compression requirements. Therefore, these technologies should be considered consistent with green-extraction principles when they reduce hazardous solvents, improve resource efficiency, or intensify processing, but claims of environmental superiority require quantitative evidence, such as energy balances, life-cycle assessment (LCA), or comparable techno-environmental analysis. In the absence of such evidence, environmental benefits should be described as potential rather than demonstrated. In phenolic-rich matrices, extraction selectivity becomes even more critical due to the structural diversity and variable stability of phenolic compounds. Vacuum-MAE of pomegranate peels generated high total phenolic content accompanied by strong radical scavenging capacity [152], whereas pressurized liquid extraction (PLE) of chokeberry by-products produced total phenolic contents exceeding 100 mg gallic acid equivalents (GAE)/g dry matter (dm) together with elevated FRAP values [156]. A direct comparison of the three extraction techniques further illustrates the importance of distinguishing individual performance indicators: PLE, MAE, and ultrasound-assisted extraction (UAE) yielded total polyphenol contents of 101.3, 94.8, and 79.9 mg GAE/g dm, respectively, whereas total monomeric anthocyanin contents were 19.3, 24.0, and 23.2 mg cyanidin-3-glucoside equivalents (C3GE)/g dm. Thus, the method providing the highest total polyphenol content did not maximize total monomeric anthocyanin content (Table 2). Importantly, antioxidant outcomes measured by 2,2-diphenyl-1-picrylhydrazyl (DPPH), 2,2′-azino-bis(3-ethylbenzothiazoline-6-sulfonic acid) (ABTS), and oxygen radical absorbance capacity (ORAC) assays differed depending on the extraction technique, indicating that solvent composition and extraction severity modulate not only the amount and profile of recovered phenolic compounds but also the qualitative antioxidant profile of the extract. Thus, process intensity and solvent polarity influence the spectrum of phenolics recovered, with direct implications for their functional performance. Importantly, coriander seeds represent a conventional food raw material rather than a food by-product; however, they provide a relevant example of how modern green extraction strategies can be applied to enhance the selective recovery of bioactive compounds. In particular, the combination of UAE with NADES illustrates the potential of integrated, sustainable extraction approaches to improve selectivity and recovery efficiency. Under optimized NADES-assisted conditions, chlorogenic acid and its isomer reached concentrations of 453.90 and 537.42 µg/g, respectively, demonstrating the effectiveness of this combined approach for targeted phenolic recovery [154], and showing that solvent system engineering can enhance the recovery of specific target molecules. Such approaches enable a transition from generalized phenolic enrichment toward the targeted recovery of defined bioactive compounds with higher added value and clearer functional positioning, thereby increasing the precision and strategic relevance of waste-derived ingredient development.

Anthocyanin-rich food matrices further illustrate the potential of modern and more sustainable extraction technologies, while not necessarily representing conventional food waste or food by-products. For instance, both PLE and UAE-assisted extraction achieved high analytical recoveries of 97.1–101.9% for anthocyanins from purple corn powder, with PLE additionally providing lower detection limits and higher throughput [159]. Although purple corn powder is a conventional food-derived material rather than a food by-product, this example is intentionally included to demonstrate the applicability of pressurized extraction systems as modern and potentially more resource-efficient approaches for the recovery and analysis of polar pigments. Similarly, DES–UAE applied to red cabbage extracts demonstrated that antioxidant properties and anthocyanin stability were strongly dependent on DES composition and storage temperature [160]. Red cabbage likewise represents a conventional food raw material rather than a food waste stream; nevertheless, its inclusion highlights the potential of combining green extraction technologies, such as UAE and DES, to improve the selective recovery while simultaneously considering the stability and functional properties of sensitive anthocyanins.These findings demonstrate that maximizing extraction efficiency does not guarantee long-term functional performance, as anthocyanin stability is strongly influenced by pH, temperature, oxygen exposure, and matrix interactions during storage and subsequent formulation. Importantly, increased extraction severity does not inherently translate into improved functional quality. This is exemplified by pomegranate seed oil, where higher extraction yield coincided with reduced phenolic content and diminished antioxidant performance [155], possibly due to degradation of sensitive compounds or co-extraction of non-active lipid fractions that dilute bioactive components. From a technological perspective, each extraction approach presents distinct operational advantages and constraints. UAE is relatively adaptable to continuous processing and operates at moderate temperatures with short extraction times, although cavitation efficiency may decrease at larger volumes, potentially affecting reproducibility. The effectiveness of cavitation-based extraction was recently demonstrated for lemon-processing by-products. Under optimized hydroalcoholic solvent conditions, UAE increased the recovery of total flavanones, hydroxycinnamic acids, and total polyphenols by 25.9%, 10.3%, and 20.5%, respectively, compared with conventional extraction [161]. Hydrodynamic cavitation further increased total polyphenol recovery by 5.2% relative to UAE and was identified as a promising approach for scalable recovery of phenolic compounds from citrus by-products. PLE ensures highly reproducible extraction cycles with precise control of temperature and pressure, yet requires high-pressure equipment and elevated temperatures, increasing operational costs and thermal energy input. NADES represent a promising green solvent platform owing to tunable polarity and low volatility, reducing reliance on conventional organic solvents; however, high viscosity, mass transfer limitations, challenges in solvent recovery and downstream purification, as well as regulatory acceptance, remain barriers to widespread industrial implementation. Across all studies, extraction efficiency is primarily governed by solvent polarity, solid-to-solvent ratio, temperature-time interplay, and applied energy intensity. Across all studies, extraction efficiency is primarily governed by solvent polarity, solid-to-solvent ratio, temperature-time interplay, and applied energy intensity. Advanced extraction technologies can reduce processing time and solvent requirements relative to conventional methods, but these advantages do not necessarily translate into a lower overall environmental burden. Their sustainability should therefore be assessed at the process-system level, considering energy demand, solvent and water requirements, downstream processing, scale, and throughput. Accordingly, rational process design for food by-product valorization should integrate extraction yield, selectivity, compound stability, scalability, resource efficiency, and environmental performance within a coherent, application-oriented framework. Where quantitative environmental assessments are unavailable, claims of sustainability should remain cautious and be framed as potential rather than demonstrated benefits. In the European Union and the United States, waste-derived extracts may be subject to jurisdiction-specific requirements concerning novel food authorization, generally recognized as safe (GRAS) status, solvent residues, contaminant thresholds, and traceability requirements. Although a detailed regulatory discussion is beyond the scope of this section, these aspects are critical for translating laboratory-scale extraction strategies into commercially viable and compliant food ingredients and will be addressed in the subsequent chapter (Section 7.2).

## 6. Encapsulation of By-Product-Derived Phytochemicals: Efficiency, Stability, and Bioaccessibility

Following the discussion of extraction performance in Section 5, this section evaluates the stabilization of recovered phytochemicals through encapsulation. Whereas Section 4 describes the principles and characteristics of the available techniques, the present section compares experimental outcomes in terms of encapsulation efficiency, compound retention, storage stability, and gastrointestinal bioaccessibility. Table 3 summarizes representative systems based on by-product-derived bioactives and related plant extracts, with particular attention to the effects of carrier composition and processing conditions. 

Among the available techniques, spray drying remains one of the most widely applied approaches because of its scalability, relatively low production cost, and compatibility with industrial food processing (Table 3). Carbohydrate-based wall materials, particularly maltodextrin and gum arabic, are frequently employed either individually or in combination [162,163,164,165,166], while plant- and dairy-derived proteins are increasingly incorporated to improve encapsulation performance and functional properties [120,167,168,169,170]. For example, red cabbage anthocyanins encapsulated in maltodextrin by spray drying achieved an encapsulation efficiency (EE) of 96.36% and a process yield of 99.4%, with an average particle size of 10.27 ± 2.8 µm, while also exhibiting improved thermal stability and an extended half-life [162]. Similarly, spray-dried purple corn extract encapsulated using maltodextrin, soy protein isolate, and gum arabic showed EE values of approximately 95–98%, although process yield varied substantially among formulations, highlighting the strong influence of wall-material composition and drying conditions on encapsulation performance [164]. In *Lonicera caerulea* pomace extract, encapsulation efficiencies ranged from 86.53 to 97.27% for phenolic compounds and from 88.17 to 96.05% for anthocyanins, while retention after 90 days of storage at 25 °C ranged from approximately 89.8 to 97.2%, demonstrating the protective capacity of maltodextrin- and protein-based matrices during storage [167]. These findings indicate that the performance of encapsulation systems depends strongly on carrier composition and processing conditions; therefore, the observed advantages should be interpreted as formulation- and process-specific rather than as universal benefits of a particular encapsulation technique.

**Table 3:** Overview of microencapsulation systems developed for bioactive compounds recovered from food processing by-products and non-edible plant materials.

Core Material	Wall Material (Major)	Complementary Polymer	Technique	Main Findings	**Ref.**
Black carrot anthocyanins	Potato protein isolate (83.6% crude protein)	Gum arabic; guar gum; inulin; maltodextrin; pectin; soluble fiber	Double emulsion (W/O/W) → complex coacervation → spray drying	Potato protein–polysaccharide systems effectively stabilized black carrot anthocyanins. Formulations containing gum arabic and pectin showed particularly promising properties for improving anthocyanin stability and encapsulation performance; potato protein–guar gum microcapsules exhibited the lowest moisture content.	[120]
Purple tea phenolic compounds	Sodium alginate	Calcium chloride	Vibrating-nozzle ionotropic gelation	EE: 84%. Microencapsulation preserved the antioxidant properties of the phenolic-rich extract and enabled its successful incorporation into a milky-tea model.	[171]
Red cabbage anthocyanins	Maltodextrin (DE 10–12)	–	Spray drying	Particle size: ~10.27 µm; EE: 96.36%; process yield: 99.4%; DPPH: 66.9%; ABTS: 71.76%. Encapsulation markedly improved thermal stability and extended anthocyanin half-life.	[162]
Raspberry pomace anthocyanins	Gum arabic	*Opuntia ficus-indica* mucilage	Spray drying	Solubility: 87.7% (GA), 70.1% (M), and 80.8% (GA+M); hygroscopicity: 1.17–1.25%. Gum arabic improved solubility, whereas mucilage increased water-holding capacity.	[163]
Onion skin flavonoids	Thermally treated starch	–	Freeze drying and spray drying	EE was substantially higher for freeze-dried microcapsules (66.6–78.0%) than for spray-dried systems (10.1–14.1%). Flavonoid retention after 8 weeks reached 89.4–98.8%.	[91]
Black carrot anthocyanins	Pea and rice protein isolates	Fructooligosaccharides (FOS)	Double emulsion (W/O/W) → complex coacervation → spray drying	Gastric solubility: 76.19%; intestinal solubility: 80.79%; intestinal bioaccessibility: 50.34%; estimated half-life: 364.8 days.	[168]
Cranberry phenolic compounds	Sodium alginate	Whey, pea, soy, pumpkin, sunflower, or rice protein isolates	Ionotropic gelation	EE: 53.2–71.8%. Whey-protein-containing microcapsules provided the highest estimated in vitro gastrointestinal bioaccessibility, whereas sunflower and pumpkin protein systems showed high phenolic content and antioxidant activity.	[172]
Purple corn cob extract	Maltodextrin; soy protein isolate	Gum arabic	Spray drying	EE: approximately 95–98%; TPC: approximately 2–3 mg GAE/g microcapsules. The obtained powders showed good stability and pH-sensitive properties.	[164]
*Lonicera caerulea* pomace extract	Maltodextrin	Soy protein isolate	Spray drying	EE for phenolic compounds: 86.53–97.27%; EE for anthocyanins: 88.17–96.05%; retention after 90 days at 25 °C: approximately 89.8–97.2%.	[167]
Calamansi peel and seed extract	Maltodextrin	Gum arabic	Spray drying	Yield: 39.57–54.73%; EE: 89.85–91.84%; TPC: 12.37–21.20 mg GAE/g; water activity: 0.15–0.22; solubility: 89.92–93.33%.	[165]
Grape skin anthocyanins	Pea or rice protein isolate	Inositol hexaphosphate (IP6)	Double emulsion (W/O/W) → complex coacervation → spray drying	Maximum anthocyanin retention: 94.4%. Increasing the wall-to-emulsion ratio produced smaller and more uniform particles, although retention decreased.	[168]
Saskatoon berry pomace polyphenols + probiotic bacteria	Pea protein isolate	Gum arabic	Spray drying/co-encapsulation	EE: 72.6% for polyphenols and 94.4% for probiotic bacteria. Intestinal bioaccessibility of TPC reached approximately 63.6%.	[169]
Grape pomace phenolic extracts	Brewery-waste *Saccharomyces cerevisiae*	–	Spray drying	Yeast cells acted as biocapsules for grape-pomace phenolics. Retention was high and encapsulation improved phenolic bioaccessibility compared with free extracts.	[173]
Sugar maple leaf phenolic extract	Maltodextrin	Gum arabic	Spray drying and freeze-drying	EE >95%; solubility >90%; intestinal bioaccessibility >43%; moisture content: 2.26–4.38%.	[166]
Tomato pomace extract	Whey protein concentrate, gelatin, or apricot kernel protein, in native or hydrolyzed form	–	Spray drying	Production yield: 52.90–85.16%; apricot kernel protein provided EE of 79.45–83.21%, TPC of 2.18–2.43 mg GAE/g DM, DPPH activity of 48.73%, and ABTS activity of 58.32%.	[170]
Peanut skin procyanidins	Inulin	Whey protein	Encapsulation using inulin–whey protein matrices	Increasing the whey-protein/inulin ratio increased EE to >98%, enhanced thermal stability, reduced particle size and porosity, and modified procyanidin release during simulated digestion.	[174]

Abbreviations: W/O/W—water-in-oil-in-water double emulsion; EE—encapsulation efficiency; FOS—fructooligosaccharides; TPC—total phenolic content; GAE—gallic acid equivalents; ABTS—2,2′-azinobis-(3-ethylbenzothiazoline-6-sulfonic acid) radical cation decolorization assay; DPPH—2,2-diphenyl-1-picrylhydrazyl radical scavenging assay; t½—half-life (time required for 50% degradation under specified storage conditions); DE—dextrose equivalent; WHC—water-holding capacity; GA—gum arabic; M—mucilage; IP6—inositol hexaphosphate; WPC—whey protein concentrate; DM—dry matter.

Yet, high encapsulation efficiency should not be interpreted as a definitive indicator of functional performance. A cross-comparison of encapsulation efficiency (EE), gastrointestinal bioaccessibility, and storage stability reveals important differences among delivery systems. For example, sugar maple leaf phenolics showed EE values exceeding 95%, whereas intestinal bioaccessibility remained above 43% [166]. Similarly, the pea–rice protein/fructooligosaccharide (FOS) system developed for black carrot anthocyanins provided an intestinal bioaccessibility of 50.34% despite its high storage stability, including an estimated half-life of 364.8 days [168]. These findings indicate that efficient initial entrapment does not necessarily translate into complete bioaccessibility under simulated gastrointestinal conditions.

This distinction is also evident in berry-derived systems. In Saskatoon berry pomace co-encapsulation, EE reached 72.6% for polyphenolic compounds and 94.4% for probiotic bacteria, whereas the intestinal bioaccessibility of total phenolic compounds was approximately 63.6% [169]. Cranberry phenolic compounds encapsulated in protein–alginate matrices showed moderate EE values of 53.2–71.8%, while estimated in vitro gastrointestinal bioaccessibility ranged from 32.77% to 45.89%, depending on the protein isolate used [172]. These results reinforce the concept that EE primarily describes the initial retention of a bioactive within the carrier, whereas gastrointestinal bioaccessibility is additionally governed by matrix disintegration, diffusion, interactions between the core and wall materials, and susceptibility of the carrier to digestive conditions. 

Yeast-based encapsulation provides another example of how carrier selection can influence functional performance. Brewery-waste *Saccharomyces cerevisiae* successfully entrapped phenolic compounds from grape pomace extracts and produced powders with low water activity, low moisture content, and suitable storage characteristics. More importantly, encapsulation increased the gastrointestinal bioaccessibility of phenolics from Bordeaux and Cabernet Sauvignon grape pomace extracts by 34.96% and 14.25%, respectively, compared with the corresponding non-encapsulated extracts [173]. Thus, evaluation of encapsulation systems should integrate initial retention, gastrointestinal bioaccessibility, and storage stability rather than relying on EE alone. 

However, the values summarized across these studies should not be interpreted as a direct ranking of carrier systems. Differences in extract composition, core-to-wall ratio, analytical procedures, and simulated digestion protocols limit the comparability of both EE and bioaccessibility. Moreover, these percentages describe different stages of delivery and may use different calculation bases. A system with higher EE may retain more compound initially without providing a larger bioaccessible amount after digestion. More informative comparisons would therefore quantify the amount of bioactive compound remaining bioaccessible per serving after processing and storage, using matched non-encapsulated controls and clearly defined digestion conditions. For commercialization, this functional advantage must also be maintained at a practical ingredient dose without unacceptable effects on food texture, flavor, or production cost. These criteria provide a stronger basis for selecting promising formulations than maximizing EE alone.

The protective performance of protein–polysaccharide systems is closely related to intermolecular interactions within the carrier matrix. Electrostatic interactions and hydrogen bonding have been identified as important contributors to the stability of protein–chitosan complex coacervates, together with the physical integration of dispersed droplets within the polymeric network [175]. More broadly, proteins can provide interfacial activity, emulsifying properties, and protective barriers for encapsulated lipophilic bioactives, while structural modification or hydrolysis may further improve their functionality as wall materials [176]. Such interactions highlight the importance of wall-material composition and molecular organization in determining both encapsulation stability and release behavior. 

Ionotropic gelation with sodium alginate and calcium chloride represents an alternative mild encapsulation strategy. In purple tea polyphenol systems, vibrating-nozzle ionotropic gelation achieved an EE of approximately 84%, while encapsulated extracts retained substantial antioxidant functionality and could be successfully incorporated into a milky-tea model [171]. Although hydrogel-based systems offer mild processing conditions and effective protection of sensitive bioactives, their relatively high moisture content, particle size, and limitations associated with large-scale processing may restrict their application in some dry food products. A comparison of drying technologies further highlights process-dependent trade-offs. For onion skin flavonoids, freeze-dried microcapsules exhibited EE values of 66.6–78.0%, substantially higher than those obtained by spray drying in the same study, while flavonoid retention after eight weeks of storage remained high at 89.4–98.8% [91]. Nevertheless, freeze drying is energy-intensive, time-consuming, and comparatively costly at industrial scale. Spray drying, despite potentially lower EE for certain formulations, remains particularly attractive for large-scale valorization because of its continuous operation, high throughput, and established industrial feasibility. Future optimization should therefore focus on wall-material composition, feed properties, atomization conditions, and drying parameters to balance bioactive protection with processing efficiency. 

From a sustainability perspective, the integration of waste-derived extracts into encapsulation platforms represents a promising pathway for upgrading low-value biomass into functional food ingredients. Such approaches can reduce waste generation, extend the use of agricultural resources, and generate higher-value products without increasing demand for primary raw materials. Nevertheless, the transition from laboratory-scale demonstrations to commercial implementation requires technological scalability as well as compliance with regulatory requirements concerning ingredient status, extraction solvents, contaminants, labeling, traceability, and safety assessment. In the European Union and the United States, waste- or by-product-derived ingredients may fall within regulatory frameworks concerning novel foods, GRAS status, and other food-safety requirements, depending on their source, processing history, and intended use. A detailed discussion of these regulatory aspects is provided in Section 7.2. 

Overall, the valorization of food-processing by-products as sources of encapsulated bioactives has produced systems with high encapsulation efficiencies, substantial improvements in storage stability, and moderate-to-promising gastrointestinal bioaccessibility. However, successful encapsulation should be evaluated through an integrated framework encompassing EE, controlled release characteristics, gastrointestinal bioaccessibility, storage stability, process scalability, and formulation compatibility. Combining these technological criteria with sustainability and regulatory considerations will be essential for translating waste-derived encapsulated bioactives from proof-of-concept systems into industrially viable functional ingredients within circular food systems.

### Design and Functional Integration of Waste-Derived Bioactive Microcapsules in Foods

The valorization of food-processing by-products and plant-derived residues and non-edible fractions for the development of functional ingredients has increasingly focused on microencapsulation as a strategy to stabilize bioactive compounds and facilitate their incorporation into diverse food matrices. These materials, including leaves, flowers, pomaces, peels, and seeds, represent valuable sources of phenolic compounds, carotenoids, and other bioactive phytochemicals. Their incorporation into encapsulation systems may enhance antioxidant potential, improve storage stability, and facilitate the development of functional foods while maintaining acceptable technological and sensory properties [177,178,179]. The data summarized in Table 4 illustrate the diversity of encapsulation strategies and their functional outcomes across different food applications.

Phenolic-rich plant extracts have been successfully microencapsulated using combinations of food-grade carbohydrate- and protein-based wall materials, with spray drying and complex coacervation among the most frequently applied approaches (Table 4). For example, thyme leaf phenolic extracts encapsulated using a maltodextrin–whey protein concentrate system by spray drying and subsequently incorporated into bread increased total phenolic content and antioxidant activity while maintaining acceptable technological characteristics of the fortified product [177]. These findings demonstrate successful incorporation and preservation of phenolic-associated antioxidant capacity in the bread matrix; however, increases in total phenolic content or chemical antioxidant activity should not be interpreted as direct evidence of a physiological antioxidant effect following consumption. Similarly, sage (*Salvia officinalis)* extract was encapsulated by complex coacervation using gum arabic and whey protein concentrate followed by freeze-drying, and incorporated into frozen yogurt, resulting in improved functional properties together with favorable textural and sensory characteristics [93].

More complex wall-material combinations may provide additional protection during food processing and storage. *Hibiscus sabdariffa* extract encapsulated by spray drying using maltodextrin, inulin, and quince seed mucilage showed improved encapsulation performance and preservation of phenolic compounds and antioxidant activity. Incorporation of these microcapsules into sponge cake resulted in higher TPC and antioxidant activity during storage without significant adverse effects on sensory attributes compared with the control [178]. These results demonstrate that multicomponent wall systems can provide effective chemical stabilization while remaining compatible with thermally processed bakery products. Nevertheless, the observed retention of phenolics and antioxidant activity during storage remains a chemical and technological endpoint. It does not establish that the encapsulated compounds remain bioaccessible after gastrointestinal digestion or exert a biological effect after absorption.

Dairy products also provide suitable matrices for the incorporation of encapsulated plant bioactives. Hydroethanolic extract from *Vitex agnus-castus* L. fruit was encapsulated by complex coacervation using gum arabic and whey protein and subsequently incorporated into yogurt, improving its functional value while maintaining favorable textural and sensory properties during 20 days of storage [180]. Similarly, saffron floral by-product extracts encapsulated by ionotropic gelation using sodium alginate were successfully incorporated into yogurt, increasing total phenolic content and antioxidant capacity while maintaining physicochemical and microbiological quality during 21 days of refrigerated storage [181]. Collectively, these studies demonstrate that encapsulation can facilitate the incorporation of bioactive-rich plant by-products into bakery and dairy products while simultaneously improving functional value and protecting sensitive phytochemicals during processing and storage. Accordingly, the technological performance of encapsulated bioactives should be evaluated not only in terms of bioactive retention, but also by considering texture, color, syneresis, sensory acceptance, and microbiological stability of the final food product. From a translational perspective, these studies should therefore be regarded primarily as food-matrix and formulation-level proof-of-concept. Validation in a real food matrix is an important step beyond characterization of the encapsulated ingredient alone because it demonstrates technological compatibility under processing, storage, and sensory conditions. Nevertheless, it represents only one level of evidence in the progression from by-product-derived extract to a biologically effective functional ingredient.

**Table 4:** Application of microcapsules containing waste- and plant-derived bioactive compounds in food matrices.

Core Material	Wall Composition	Technique	Application	Key Findings	**Ref.**
Thyme leaves phenolic compounds	Maltodextrin + Whey protein concentrate	Spray drying	Bread	Increased TPC (*p* < 0.05) and DPPH radical scavenging activity (*p* < 0.05). 5% microcapsule addition did not affect crumb structure (porosity) or specific volume (*p* > 0.05). No significant effect on compressive strength of fresh crumb (day 0) or after 3 days of storage (*p* > 0.05).	[177]
Sage extract	Gum arabic + Whey protein concentrate	Complex coacervation + Freeze drying	Frozen yogurt	Microcapsules influenced textural properties: firmness, adhesiveness, and consistency. Frozen yogurt with microcapsules showed improved sensory attributes for color, flavor, and texture.	[93]
Hibiscus sabdariffa extract	Maltodextrin + Quince seed mucilage + Inulin	Spray drying	Sponge cake	No statistically significant differences between control and fortified cake in texture, taste, color, odor, and overall acceptance (*p* > 0.05). Significantly higher TPC and DPPH scavenging activity were maintained during 28 days of storage (*p* < 0.05).	[178]
*Vitex agnus-castus* L. fruit extract	Whey protein concentrate + Gum arabic	Complex coacervation + Freeze drying	Yogurt	Improved texture and appearance in sensory analysis. High overall quality maintained after 20 days of storage.	[180]
Herbal extracts (rose, borage, balm, saffron)	Gum arabic + Maltodextrin	Coacervation + Spray drying	Dark chocolate	Microcapsules affected textural properties: breaking force (N), brittleness (mm), viscosity, and Casson yield stress (Pa). Sensory attributes influenced: melting behavior, grittiness, and herbal aroma.	[182]
Saffron flower extract	Sodium alginate + CaCl_2_	Ionotropic gelation + Freeze-drying	Yogurt	Microcapsule incorporation significantly reduced syneresis during 21 days of storage at 4 °C. Increased TPC and antioxidant potential measured by FRAP, ABTS, and DPPH assays.	[179]
Carotenoid extract from carrot peel	Poloxamer 407	Nanostructured lipid carriers (NLCs)	Yogurt	Enhanced textural properties: firmness, consistency, cohesiveness, and cohesion. High carotenoid retention (>97%) and stability during 28 days at 4 °C, dark, minimal headspace. No syneresis observed during storage.	[131]

Abbreviations: TPC—total phenolic content; DPPH—2,2-diphenyl-1-picrylhydrazyl radical scavenging assay; ABTS—2,2′-azinobis-(3-ethylbenzothiazoline-6-sulfonic acid) radical cation decolorization assay; FRAP—ferric reducing antioxidant power; CaCl_2_—calcium chloride; N—newtons (unit of force); mm—millimeters; Pa—pascals; NLCs—nanostructured lipid carriers.

The results underscore the potential of encapsulation systems to enhance both the functional and technological performance of liquid and semi-solid foods, in which high water activity and phase instability may otherwise compromise bioactive retention. Beyond phenolic compounds, carotenoid-rich extracts recovered from carrot peels have been successfully incorporated into nanostructured lipid carriers (NLCs) containing Poloxamer 407 and subsequently used to fortify yogurt [131]. During 28 days of refrigerated storage at 4 °C, the fortified yogurt maintained stable texture, viscosity, and color, while pH, titratable acidity, and syneresis followed trends comparable to those of the control. Importantly, carotenoid losses were limited to approximately 0.2–2.8%, corresponding to retention of approximately 97.2–99.8%, demonstrating the strong protective capacity of the lipid-based carrier under refrigerated storage conditions. These findings indicate that NLCs can effectively protect oxidation-sensitive lipophilic compounds while maintaining the physicochemical stability of the food matrix. 

Overall, the available evidence reveals a clear pattern: core characteristics, wall-material composition, and encapsulation technique collectively determine bioactive retention and functional performance. Protein–polysaccharide combinations, including whey protein–gum arabic and protein–maltodextrin systems, frequently improve physical and chemical stability and may influence bioactive release, whereas carbohydrate-based matrices alone may be sufficient for certain thermally processed foods or predominantly hydrophilic compounds. Spray drying and freeze drying remain among the most frequently applied techniques in the studies considered, while complex coacervation provides additional opportunities for tailoring interfacial organization and carrier stability. 

Protein–polysaccharide complex coacervates may also serve as functional stabilizing structures in emulsion-based delivery systems. For example, insect protein–chitosan complex coacervates used as stabilizers of β-sitosterol-loaded Pickering emulsions provided bioactive retention of 56–91%, with emulsion stability associated with electrostatic interactions, hydrogen bonding, and physical integration of oil droplets within the polymeric matrix [175]. These findings illustrate how the molecular organization of multicomponent wall systems can influence both carrier integrity and bioactive retention. Lipid-based carriers are particularly suitable for hydrophobic compounds because they can protect oxidation-sensitive bioactives and facilitate their incorporation into aqueous or semi-solid food matrices. Importantly, although many studies report high initial retention or encapsulation efficiency, these parameters alone do not determine the long-term functional performance of the resulting food product. The extent to which encapsulated compounds remain stable during storage, are released during digestion, and become bioaccessible depends strongly on interactions among the bioactive compound, carrier material, and food matrix. 

Moreover, multicomponent wall systems, although potentially advantageous in terms of stability and functionality, may increase formulation complexity and production costs at industrial scale. Translation of laboratory-scale systems into commercially viable applications therefore requires consideration of scalability, energy demand, process efficiency, ingredient cost, and compatibility with the final food matrix. Encapsulation systems should consequently be optimized not only for maximum bioactive retention but also for technological feasibility, gastrointestinal performance, storage stability, and suitability for the intended food application.

In conclusion, microencapsulation of bioactive compounds derived from food-processing by-products represents a promising strategy to combine functional ingredient delivery with sensory and structural quality across diverse food matrices. The reviewed studies demonstrate that both polar and non-polar phytochemicals can be effectively stabilized and incorporated; however, success depends on the rational design of wall materials, encapsulation techniques, and compatibility with the target food system. Future research should integrate systematic evaluation of gastrointestinal bioaccessibility, release kinetics, and long-term stability to ensure that high encapsulation efficiency translates into improved functional performance in commercially relevant products.

## 7. Prospects and Challenges

Despite the significant progress achieved in the extraction, encapsulation, and application of bioactive compounds recovered from food-industry by-products, several scientific, technological, economic, and regulatory challenges still hinder their widespread industrial implementation. Major limitations include the compositional variability of by-product streams, difficulties in standardizing extraction and encapsulation processes, scale-up constraints, limited information on long-term storage stability and gastrointestinal fate, and the need to ensure sensory compatibility with complex food matrices. In addition, the safety, regulatory status, and batch-to-batch consistency of waste-derived ingredients require careful consideration before commercial application. Addressing these challenges, together with rigorous techno-economic and environmental assessment, is essential for translating laboratory-scale findings into commercially viable functional foods and for fully exploiting the potential of by-product valorization within a circular economy framework.

### 7.1. Technological and Economic Limitations

Despite the rapid advances in encapsulation technologies, several challenges still limit their large-scale implementation in the food industry. One of the major concerns is the stability of bioactive compounds, which are highly susceptible to degradation induced by oxygen, light, temperature, and pH fluctuations during processing and storage [27,47].

Another important limitation is the scalability of encapsulation processes. Many technologies that have demonstrated excellent performance at the laboratory scale, including electrospraying, microfluidics, and certain nanotechnology-based systems, remain difficult to implement industrially because of low production throughput, process complexity, and high operating costs [56,183].

The economic feasibility of encapsulation also remains a significant challenge. Production costs are often increased by the use of high-purity encapsulating materials and energy-intensive processes such as freeze drying and spray drying [44,47]. Recent techno-economic and life-cycle assessment of pomegranate peel extract encapsulation confirmed the strong scale dependence of production costs and identified freeze drying as the major environmental hotspot of the process [184]. Furthermore, the commercialization of encapsulated ingredients derived from food-industry by-products is complicated by the inherent variability of raw materials. Differences in botanical origin, seasonal variations, processing conditions, and storage practices can substantially affect the composition and concentration of recovered phytochemicals, making the standardization of extraction and encapsulation processes particularly challenging [18,185]. 

### 7.2. Regulation and Consumer Acceptance

The application of encapsulation technologies in food systems is subject to strict regulatory requirements. In the European Union, novel ingredients and production technologies may fall under the scope of the Regulation (EU) 2015/2283, whereas in the United States, the safety of food ingredients is primarily evaluated according to the Generally Recognized as Safe (GRAS) framework [186,187,188].

The use of bioactive compounds recovered from food-industry by-products may also require compliance with regulations concerning contaminant levels, traceability, labeling requirements, and quality assurance. These regulatory aspects can significantly influence the translation of laboratory-scale technologies into commercially viable products [188].

Consumer acceptance represents another important factor affecting the market success of encapsulated functional foods. Although encapsulation may reduce undesirable sensory attributes associated with certain phytochemicals, including bitterness and astringency, the incorporation of encapsulated ingredients may still influence the texture, appearance, and overall sensory quality of food products [189]. Therefore, future product development should focus not only on improving the stability and bioavailability of bioactive compounds but also on maintaining high consumer acceptability.

### 7.3. Future Research Directions

Future research should prioritize the development of sustainable and food-grade encapsulating materials, including polysaccharides, proteins, and biopolymers obtained from renewable resources and food-industry by-products, thereby supporting circular economy strategies [66,185].

Particular attention should be devoted to the design of delivery systems with improved gastrointestinal stability and controlled release properties, enabling site-specific or modulated release of bioactive compounds under defined gastrointestinal conditions [28]. The integration of encapsulation technologies with active and intelligent packaging systems capable of responding to environmental stimuli also represents a promising research direction [190].

Furthermore, future studies should move beyond in vitro characterization and focus on in vivo assessment of bioavailability, bioactivity, and long-term safety. Additional efforts are required to improve process scalability, economic feasibility, and life-cycle sustainability assessments to facilitate the industrial implementation of encapsulated ingredients recovered from food-industry by-products.

Finally, the integration of green extraction technologies with encapsulation processes within a biorefinery framework offers considerable potential for converting low-value agro-industrial residues into high-value functional ingredients and next-generation functional foods [18,189].

## 8. Materials and Methods

This structured narrative review was based on a literature search conducted across the Scopus, Web of Science, PubMed, and Google Scholar databases. The search strategy employed combinations of Boolean operators (AND, OR) with the following key terms: (i) encapsulation technologies (encapsulation, microencapsulation, nanoencapsulation, delivery systems); (ii) source materials (food by-products, food waste, agro-industrial by-products, food processing residues); (iii) target compounds (phytochemicals, bioactive compounds, polyphenols); and (iv) sustainability aspects (green extraction, sustainable extraction, valorization, functional foods, and bioavailability). The initial literature search was completed in February 2026 and was subsequently updated during manuscript revision to include relevant studies published up to September 2026.

The inclusion criteria were limited to peer-reviewed articles and reviews published in English up to September 2026. While the primary emphasis was placed on recent advancements from the last decade (2016–2026) to ensure the inclusion of current scientific evidence, seminal earlier publications were selectively included to provide essential historical context and describe fundamental concepts. Exclusion criteria included non-peer-reviewed content, papers with unavailable full texts, and studies not directly related to food-grade applications of food-industry by-products or their derived bioactive compounds.

Records retrieved from multiple databases were cross-checked, and duplicate publications were removed. Titles and abstracts were initially screened for relevance to the scope of the review, followed by full-text assessment of potentially relevant studies. Final selection was based on relevance to the predefined thematic areas, availability of sufficient methodological information, and contribution of pertinent qualitative or quantitative evidence to the objectives of the review. Studies included in Table 2, Table 3 and Table 4 were selected as representative and quantitatively informative examples of the major extraction, encapsulation, and food-application strategies discussed in the review. Preference was given to studies reporting clearly defined processing conditions and measurable outcomes related to extraction performance, encapsulation characteristics, stability, gastrointestinal bioaccessibility, or functional performance in food matrices. As this study was designed as a structured narrative review rather than a systematic review, no formal PRISMA-based screening procedure was applied. Generative artificial intelligence tools were utilized strictly for English language editing and the refinement of graphical illustrations to improve clarity and visual communication. No AI tools were employed for data collection, analysis, interpretation, or the formulation of scientific conclusions. The authors have critically reviewed and verified all AI-assisted content and assume full responsibility for the integrity and accuracy of the final manuscript.

## 9. Conclusions

Encapsulation is an important strategy for improving the stability, handling, and gastrointestinal delivery of bioactive compounds intended for functional food applications.

Despite their high health-promoting potential, bioactive compounds exhibit significant technological limitations, such as limited gastrointestinal bioaccessibility and, in some cases, low systemic bioavailability, sensitivity to environmental factors, and a negative impact on the sensory characteristics of products. These limitations justify the use of encapsulation techniques. 

By-products from the food industry represent a valuable and underutilized source of bioactive compounds. Their valorization aligns with the concepts of the circular economy and sustainable development. 

The judicious selection of encapsulation techniques and coating materials is paramount for enhancing process efficiency and optimizing the properties of the final product, encompassing stability, release kinetics, and compatibility with the food matrix. 

High encapsulation efficiency (EE) does not necessarily indicate functionality, as other factors, including gastrointestinal bioaccessibility, systemic bioavailability, bioactivity, and stability during storage and digestion, must also be considered. Protein–polysaccharide combinations may provide complementary interfacial and matrix-stabilizing functions that improve bioactive retention and modulate release. 

In several model food systems, encapsulated bioactives have been incorporated into baked goods, dairy products, and confectionery without major adverse effects on sensory or textural properties. 

The industrial implementation of encapsulation technologies requires simultaneous consideration of technological performance, economic feasibility, process scalability, regulatory compliance, environmental impact, and consumer acceptance.

## Figures and Tables

**Table 1:** Overview of food-grade encapsulation techniques used for the stabilization of bioactive compounds recovered from food-industry by-products.

Encapsulation Technique	Main Principle	Advantages	Limitations	Typical Applications	**Advantages for By-Product Valorization**
Ionic gelation	Ion-induced biopolymer gelation	Mild conditions; biodegradable	Porosity; compound leakage	Anthocyanins, phenolics, probiotics	Protection of heat-sensitive extracts
Extrusion	Droplet formation and solidification	Simple; low cost	Large particles	Phenolics, probiotics	Food-grade beads and capsules
Electrospraying	Electric-field particle formation	Uniform particles; low heat	Low throughput; scale-up	Phenolics, vitamins	Suitable for thermolabile compounds
Spray drying	Atomization and rapid drying	Low cost; scalable	Heat and oxidation sensitivity	Phenolics, carotenoids, essential oils	Stable powders from liquid extracts
Freeze drying	Freezing and ice sublimation	High bioactive retention	Expensive; time-consuming	Phenolics, anthocyanins	Preservation of thermolabile compounds
Fluidized-bed coating	Particle coating during fluidization	Additional protection	Process complexity	Coated powders, flavors	Improved storage stability
Antisolvent precipitation	Solute precipitation by antisolvent	Small particles; improved dissolution	Solvent removal; aggregation	Flavonoids, phenolics	Improved dispersibility and bioaccessibility
Supercritical fluid encapsulation	Particle formation using scCO_2_	Low solvent residues; mild conditions	High equipment cost	Phenolics, carotenoids, essential oils	Integration with green processing
Conventional emulsions and nanoemulsions	Oil–water dispersion	Improved dispersibility and bioaccessibility	Physical instability	Phenolics, carotenoids, essential oils	Incorporation into aqueous foods
Double emulsions	Multiple W/O/W or O/W/O compartments	Co-encapsulation; controlled release	Structural instability; complexity	Anthocyanins, phenolics, probiotics	Delivery of compounds with different solubilities
Pickering emulsions	Particle-stabilized interface	High stability; clean-label potential	Scale-up challenges	Phenolics, carotenoids, essential oils	By-products as stabilizers and functional ingredients
Complex coacervation	Association of oppositely charged biopolymers	High EE; controlled release	pH and ionic-strength sensitivity	Anthocyanins, phenolics, essential oils	Protection using protein–polysaccharide systems
Liposomes	Phospholipid bilayer vesicles	Encapsulate hydrophilic and lipophilic compounds	Oxidation; leakage; cost	Phenolics, carotenoids, vitamins	Improved delivery and dispersibility
SLNs	Solid lipid matrix	Protection; controlled release	Low loading; compound expulsion	Phenolics, carotenoids	Protection of lipophilic bioactives
NLCs	Mixed solid–liquid lipid matrix	High loading; reduced expulsion	Complex formulation	Phenolics, carotenoids, vitamins	Stabilization of concentrated extracts
Cyclodextrin inclusion complexes	Host–guest inclusion	Improved solubility and stability	Limited cavity size	Flavonoids, essential oils	Improved stability and bioaccessibility
Layer-by-layer (LbL) systems	Sequential multilayer coating	Tunable protection and release	Limited scalability	Phenolics, lipid carriers	Improved storage stability

Abbreviations: CO_2_—carbon dioxide; EE—encapsulation efficiency; LbL—layer-by-layer; NLCs—nanostructured lipid carriers; O/W/O—oil-in-water-in-oil; scCO_2_—supercritical carbon dioxide; SLNs—solid lipid nanoparticles; W/O/W—water-in-oil-in-water.

## Data Availability

No new data were created or analyzed in this study. Data sharing is not applicable to this study.

## Abbreviations

The following abbreviations are used in this manuscript:

ABTS	2,2′-azinobis(3-ethylbenzothiazoline-6-sulfonic acid) radical cation decolorization assay
AI	artificial intelligence
C3GE	cyanidin-3-glucoside equivalents
CAGR	compound annual growth rate
ChCl	choline chloride
CO_2_	carbon dioxide
DE	dextrose equivalent
DES	deep eutectic solvent
DM	dry matter
DPPH	2,2-diphenyl-1-picrylhydrazyl radical scavenging assay
EAE	enzyme-assisted extraction
EE	encapsulation efficiency
EFSA	European Food Safety Authority
EtOH	ethanol
EU	European Union
FAO	Food and Agriculture Organization of the United Nations
FDA	U.S. Food and Drug Administration
FOS	fructooligosaccharides
FRAP	ferric reducing antioxidant power
GA	gum arabic
GAE	gallic acid equivalents
GRAS	Generally Recognized As Safe
IP6	inositol hexaphosphate
LbL	layer-by-layer
LEO	lemon essential oil
LP	lemon pigments
M	mucilage
MAC	maceration
MAE	microwave-assisted extraction
MAHD	microwave-assisted hydrodistillation
NADES	natural deep eutectic solvent
NCDs	noncommunicable diseases
NLCs	nanostructured lipid carriers
O/W/O	oil-in-water-in-oil
ORAC	oxygen radical absorbance capacity
PLE	pressurized liquid extraction
PLGA	poly(lactic-co-glycolic acid)
RSD	relative standard deviation
SAA	supercritical assisted atomization
scCO_2_	supercritical carbon dioxide
SFE	supercritical fluid extraction
SLNs	solid lipid nanoparticles
SWE	subcritical water extraction
TMA	total monomeric anthocyanins
TP	total polyphenols
TPC	total phenolic content
UAE	ultrasound-assisted extraction
VMAAE	vacuum microwave-assisted aqueous extraction
W/O/W	water-in-oil-in-water
WHC	water-holding capacity
WPC	whey protein concentrate
t½	half-life
τmax	maximum oxidation induction time
